# Gain‐of‐Function Engineering of the NifEN Scaffold Into a Self‐Assembling Nitrogenase

**DOI:** 10.1002/anie.8683437

**Published:** 2026-09-21

**Authors:** Yimo Yang, Chi Chung Lee, Steve Ortiz, Markus W. Ribbe, Yilin Hu

**Affiliations:** ^1^ Department of Molecular Biology and Biochemistry University of California Irvine California USA; ^2^ Department of Chemistry University of California Irvine California USA

**Keywords:** M‐cluster, NifEN, nitrogenase, P‐cluster, protein engineering

## Abstract

Biological nitrogen fixation by Mo‐nitrogenase is a complex process requiring coordinated electron transfer through specialized metal clusters. NifEN, an α_2_β_2_ heterotetrameric homolog of the nitrogenase catalytic component NifDK, naturally serves as a maturation scaffold but exhibits only trace catalytic activity due to its “imperfect” cluster environments. In this study, we systematically engineered NifEN to reconstruct the structural features of NifDK, specifically targeting the P→M cluster electron relay and the active‐site M‐cluster environment. Specifically, we developed a series of variants, culminating in NifEN^P/M_S2B^, which incorporates a restored P‐cluster species and a refined M‐cluster coordination site and S2B‐associated belt region. Spectroscopic analysis confirmed the successful installation of the target clusters. Moreover, activity assays and nanoSIMS analysis demonstrated that NifEN^P/M_S2B^ supported ATP‐dependent N_2_ reduction both in vitro and in vivo. Structural modeling further identified remaining catalytic bottlenecks, particularly those in the S3A and S5A regions, highlighting the importance of precise cofactor belt organization for substrate reduction. Together, these findings establish NifEN as a self‐assembling, catalytically competent nitrogenase scaffold that bypasses the need for complex cofactor transfer steps, providing a streamlined platform for mechanistic studies and engineering efforts of biological nitrogen fixation.

## Graphical abstract

From scaffold to enzyme: Systematic engineering of the cofactor maturation scaffold NifEN reconstructs the P→M electron relay along with cofactor coordination and proton gating at the S2B site, converting NifEN into a functional nitrogenase capable of ATP‐dependent N_2_ reduction both in vitro and in vivo. This self‐assembling system provides a streamlined platform for mechanistic studies and heterologous engineering of biological nitrogen fixation.

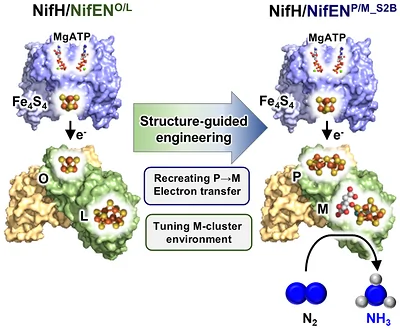

## 1 Introduction

The biological reduction of dinitrogen (N_2_) to ammonia (NH_3_) by nitrogenase is among the most challenging chemical transformations in nature. This process requires the tightly coordinated delivery of electrons and protons to a complex metallocofactor, which enables substrate reduction under ambient conditions. To date, three homologous nitrogenases (Mo‐, V‐, and Fe‐only nitrogenases) have been identified, distinguished primarily by the heterometal present in their active‐site cofactors [1, 2, 3, 4]. Each system consists of a reductase component (NifH, VnfH, or AnfH) and a catalytic component (NifDK, VnfDGK, or AnfDGK) [1, 2, 3, 4]. In the canonical Mo‐nitrogenase, the reductase component (NifH) is a homodimer that contains a subunit‐bridging [Fe_4_S_4_] cluster and a MgATP‐binding site within each subunit (Figure S1) [5, 6, 7, 8, 9, 10]. The catalytic component (NifDK) is an α_2_β_2_ heterotetramer that houses two complex metal clusters per αβ dimer: the P‐cluster ([Fe_8_S_7_]) located at the α/β interface, and the M‐cluster ([(*R*‐homocitrate)MoFe_7_S_9_C]) embedded within the α‐subunit (Figure S1) [5, 6, 7, 8, 9, 10, 11, 12, 13]. Catalysis by the Mo‐nitrogenase involves repeated cycles of association and dissociation between NifH and NifDK, which enable ATP‐dependent electron transfer from the [Fe_4_S_4_] cluster of NifH—through the P‐cluster—to the active‐site M‐cluster, where substrate reduction takes place (Figure S1) [5, 6, 7, 8, 9, 10, 11, 12, 13].

Despite extensive biochemical, spectroscopic, and structural investigations, the complexity of the Mo‐nitrogenase and, particularly, its catalytic component NifDK, continues to present a major obstacle to mechanistic analysis and engineering of nitrogen fixation. Considerable effort has been directed toward identifying simplified homologs that retain key features of nitrogenase while offering greater experimental tractability. In this context, NifEN has emerged as an especially powerful model system. An α_2_β_2_ tetrameric homolog of NifDK, NifEN contains two metal centers that correspond to those found in NifDK: an O‐cluster ([Fe_4_S_4_]), which resembles half of a P‐cluster [14], and an L‐cluster ([Fe_8_S_9_C]) [14, 15, 16], a precursor that closely resembles the M‐cluster except for the absence of Mo and homocitrate (Figure 1). Known for its central role in cofactor biosynthesis [14, 17, 18, 19, 20, 21, 22, 23, 24, 25], NifEN enables maturation of the L‐cluster to an M‐cluster through NifH/ATP‐mediated insertion of Mo and homocitrate (Figure S2a). Additionally, when loaded with an L‐cluster, NifEN is capable of supporting NifH/ATP‐dependent substrate reduction, although its catalytic activity is rather limited compared to that of NifDK (Figure S2b, left) [23, 26].

**FIGURE 1:**
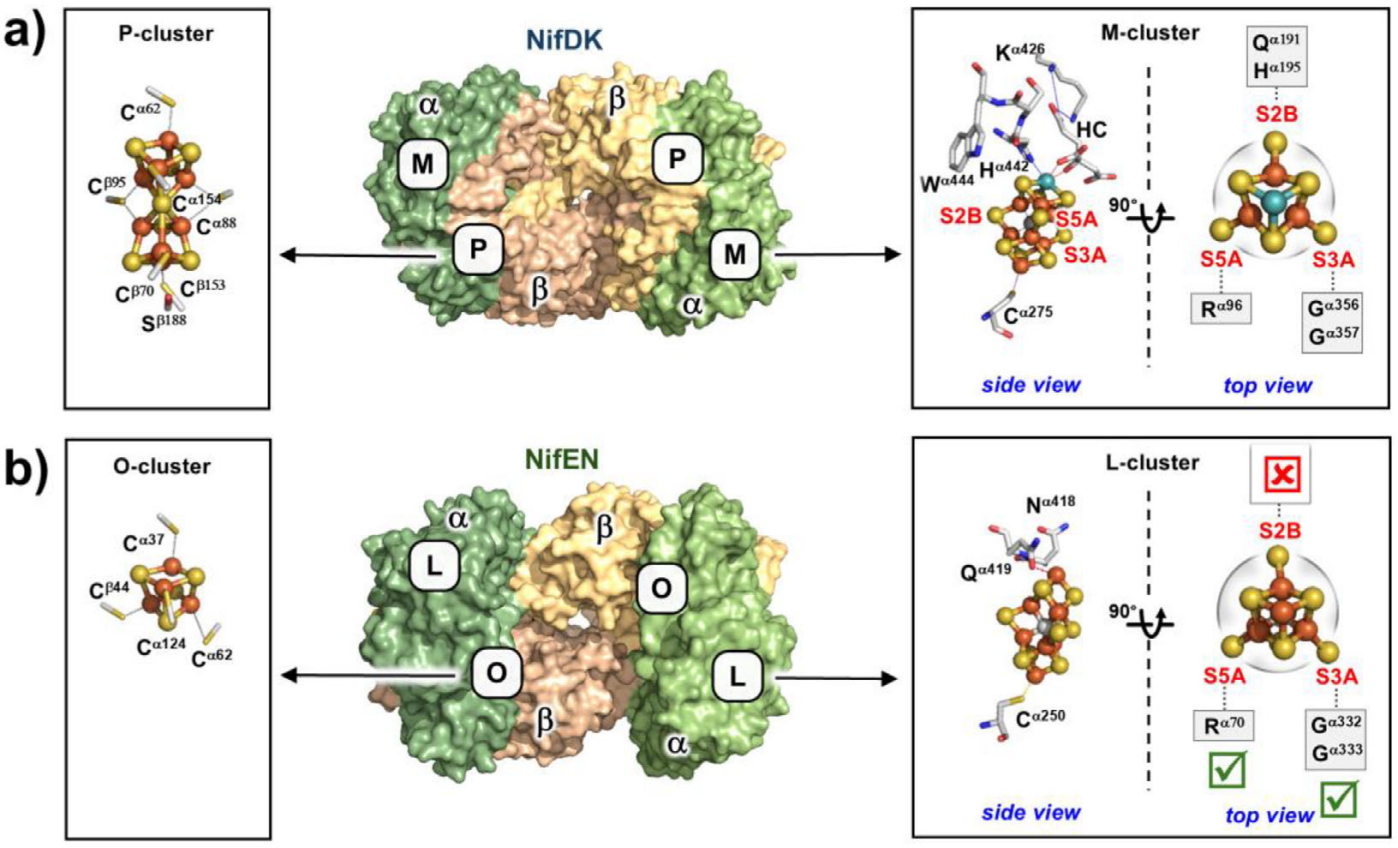
Structural comparison of the catalytic component NifDK and the maturation scaffold NifEN. (a) The α_2_β_2_‐heterotetrameric catalytic component NifDK (green/yellow) of the Mo‐nitrogenase contains a P‐cluster ([Fe_8_S_7_]) at each α/β‐subunit interface and an active‐site M‐cluster ([(*R*‐homocitrate)MoFe_7_S_9_C]) within each α‐subunit (middle). The P‐cluster is coordinated by six cysteine ligands (C^α62^, C^α88^, C^α154^, C^β70^, C^β95^, and C^β153^) and one serine residue (S^β188^) (left). The side view of the M‐cluster (le) highlights the terminal ligands C^α275^ and H^α442^, the homocitrate anchor K^α426^, and the H^α442^/W^α444^ “switch/lock” mechanism that secures the mature cofactor following insertion. The top view (right) shows that the three belt sulfurs (S2B, S3A, S5A) are positioned in close proximity to catalytically important residues Q^α191^ and H^α195^ (S2B), G^α356^ and G^α357^ (S3A), and R^α96^ (S5A). (b) A NifDK homolog, the α_2_β_2_‐heterotetrameric cofactor maturation scaffold NifEN (green/yellow) contains an O‐cluster ([Fe_4_S_4_]) at each α/β‐subunit interface and an L‐cluster ([Fe_8_S_9_C]) within each α‐subunit (middle). In contrast to NifDK, the O‐cluster of NifEN is coordinated by only four cysteine ligands (C^α37^, C^α62^, C^α124^, and C^β44^), resulting in a structurally deficient coordination environment for a complete P‐cluster (left). The side view of the L‐cluster (right) illustrates that the interior cofactor‐binding site of NifEN lacks the terminal ligand H^α442^, the H^α442^/W^α444^ “switch/lock” mechanism, and the K^α426^ homocitrate (HC) anchor that are present in NifDK. The top view (right) further demonstrates that the catalytically essential residues Q^α191^ and H^α195^, which form the proton‐gating network adjacent to the belt‐sulfur S2B in NifDK, are replaced by Lys and Asn, respectively, in NifEN, thereby restricting proton delivery to the active site. Metal center atoms (ball‐and‐stick representation) are colored as follows: Fe, orange; S, yellow; Mo, cyan; O, red; C, gray; and N, blue. Amino acid sequence alignments of the NifDK and NifEN regions highlighted in this figure are provided in Figure S3. PyMOL was used to generate this figure, using PDB entries 3U7Q [11], 7UT8 [10] and 3PDI [14].

The combination of pronounced structural homology and attenuated catalytic capability makes NifEN an attractive system for dissecting the structural features required for efficient nitrogenase catalysis. Indeed, comparisons with NifDK suggest two principal catalytic limitations of NifEN (Figures 1 and S3). First, replacement of the P‐cluster by the simpler O‐cluster could impair the electron‐transfer pathway that connects NifH to the active‐site cofactor, thereby compromising the catalytic potential of NifEN (Figures 1 and S3) [10, 11, 14]. Second, the cofactor‐binding environment of NifEN differs substantially from that of NifDK; specifically, the cofactor‐binding site is of lower affinity relative to that within NifDK due to the absence of certain ligands, and the suite of catalytically crucial residues surrounding the cofactor belt‐sulfurs is incomplete (Figures 1 and S3) [8, 11, 14, 25, 27, 28, 29, 30, 31, 32, 33, 34]. These differences reflect the role of NifEN in cofactor maturation rather than substrate reduction. Consistent with this argument, maturation of the L‐cluster into an M‐cluster [24, 35, 36]—likely accompanied by transfer of the cluster from the surface location (Figure S2b, left) to the interior cofactor‐binding site (Figure S2b, right)—results in a loss of the limited catalytic activity of NifEN [19, 22, 23]. This observation not only supports the native role of NifEN as a cofactor maturation scaffold, but also indicates that its catalytic capacity is constrained by an “imperfect” cofactor environment.

Here, we sought to determine whether nitrogenase activity could be reconstructed in NifEN through systematic restoration of the structural features found in NifDK that support electron transfer and catalysis. By engineering a series of NifEN variants that progressively reconstituted the P‐cluster environment and key structural elements surrounding the M‐cluster, we generated a P/M‐cluster‐containing NifEN variant that recapitulated the ATP‐dependent reduction of N_2_ both in vitro and in vivo when paired with the reductase NifH. These findings establish NifEN as a tractable platform for gain‐of‐function engineering of nitrogenase while providing crucial insights into the structural requirements underlying efficient biological N_2_ reduction.

## 2 Results

### 2.1 Protein Engineering

Comparative analysis of NifEN and NifDK identifies major differences at their respective P‐ and M‐cluster sites. At the P‐cluster site, NifDK coordinates the [Fe_8_S_7_] P‐cluster through six cysteine ligands (C^α62^, C^α88^, C^α154^, C^β70^, C^β95^, and C^β153^) and one Ser residue (S^β188^) [9, 10, 11], providing both stable ligation and redox flexibility required for efficient electron transfer (Figure 1a, left; Figure S3). In contrast, NifEN retains only four cysteine ligands (C^α37^, C^α62^, C^α124^, and C^β44^) [14] and instead houses a simpler [Fe_4_S_4_] O‐cluster (Figure 1b, left; Figure S3). At the M‐cluster site, NifDK uses conserved residues to anchor and orient the cofactor, including the terminal ligands C^α275^ and H^α442^ [9, 10, 11], the homocitrate anchor K^α426^ [30], and the “switch/lock” mechanism provided by H^α442^/W^α444^ [17, 37] that secures the mature cofactor upon a swap of the two residues in position (Figure 1a, right; Figure S3). In comparison, NifEN likely retains only C^α250^ as a terminal ligand, replacing the other terminal ligand with a weaker one—possibly N^α418—^for Mo coordination, while substituting the switch/lock residues and the homocitrate ligand with Asn/Gln and Arg, respectively (Figure 1b, right; Figure S3) [21]. Although this more flexible coordination could facilitate M‐cluster release during biosynthesis, it compromises cofactor stabilization and orientation. In addition, while the protein environments near the belt‐sulfurs S3A and S5B appear to be preserved, the catalytically important Q^α191^ and H^α195^ [8] adjacent to the belt‐sulfur S2B are replaced with Lys and Asn in NifEN [21], thereby restricting proton delivery for substrate reduction (Figure 1b, right; Figure S3).

The differences in P‐cluster ligation, together with the altered coordination and belt‐sulfur environment of the M‐cluster, likely account for the diminished catalytic ability of NifEN. To test this hypothesis, we used *Escherichia coli* as a heterologous expression host [38, 39, 40] to generate a series of NifEN variants representing progressive reconstruction of the NifDK metal‐center architecture (see Table S1 and Figure S4 for details of strains and proteins used in this work). The previously described wild‐type NifEN containing O‐ and L‐clusters [NifEN^O/L^] [40] served as the starting scaffold. Co‐expression of this wild‐type NifEN protein with NifH [35] and NifV [41] in the presence of molybdate generated NifEN^O/M^ upon in vivo L‐to‐M‐cluster maturation. Engineering of the electron‐transfer and active‐site environments of the wild‐type NifEN, followed by co‐expression with NifH and NifV in the presence of molybdate, yielded two P‐ and M‐cluster‐containing variants. One, designated NifEN^P/M_ΔS2B^, was designed to restore the P→M electron‐transfer pathway while retaining a deficient S2B‐site of the cofactor environment; the other, termed NifEN^P/M_S2B^, incorporated additional modifications to reconstruct the S2B‐site features of the NifDK active site.

All proteins were isolated anaerobically by immobilized metal affinity chromatography as soluble brown proteins with typical yields of 100–150 mg per 100 g wet cells. SDS–PAGE analysis confirmed the expected subunit composition of the α_2_β_2_ heterotetrameric NifEN, with the α‐ and β‐subunits exhibiting apparent molecular masses of approximately 50 and 49 kDa, respectively (Figure S5a). Metal analysis further verified the Fe and Mo contents of these NifEN species and, together with EPR data (see below), supported assignment consistent with their designed cluster compositions (Figure S5b). Despite the reduced cluster occupancy associated with heterologous expression, this series of NifEN proteins provides an effective framework for systematically evaluating how restoration of the electron‐transfer pathway and reconstruction of the active‐site environment contribute to nitrogenase catalysis.

### 2.2 Spectroscopic Characterization

Parallel‐mode EPR spectroscopy of IDS‐oxidized samples was performed to monitor P‐cluster reconstruction (Figure 2a). Neither NifEN^O/L^ nor NifEN^O/M^ exhibited the characteristic oxidized P‐cluster (P^OX^) signal observed for NifDK, consistent with the presence of an O‐cluster instead of a P‐cluster in these NifEN proteins. In contrast, both NifEN^P/M_ΔS2B^ and NifEN^P/M_S2B^ displayed a P^OX^‐like parallel‐mode feature at *g* ≈ 12, demonstrating the successful restoration of a P‐cluster‐like species in these NifEN species. Relative to the P^OX^ signal of NifDK at *g *= 11.8 [42], both NifEN^P/M_ΔS2B^ and NifEN^P/M_S2B^ displayed a slightly shifted P^OX^ signal and notably reduced signal intensities, reaching 22% and 34% of the signal intensity of the P‐cluster in NifDK, respectively. The slight shift in the *g* value indicates that the engineered P‐cluster in these NifEN variants remains electronically somewhat distinct from the native P‐cluster; whereas the reduced intensity suggests the presence of immature P*‐cluster species (i.e., paired [Fe_4_S_4_] clusters; Figure S6) and/or incompletely occupied P‐cluster sites. Thus, while the EPR data support the formation of a mature P‐cluster species in the engineered NifEN variants, they also indicate a somewhat heterogeneous cluster population that does not fully recapitulate the native P‐cluster of NifDK.

**FIGURE 2:**
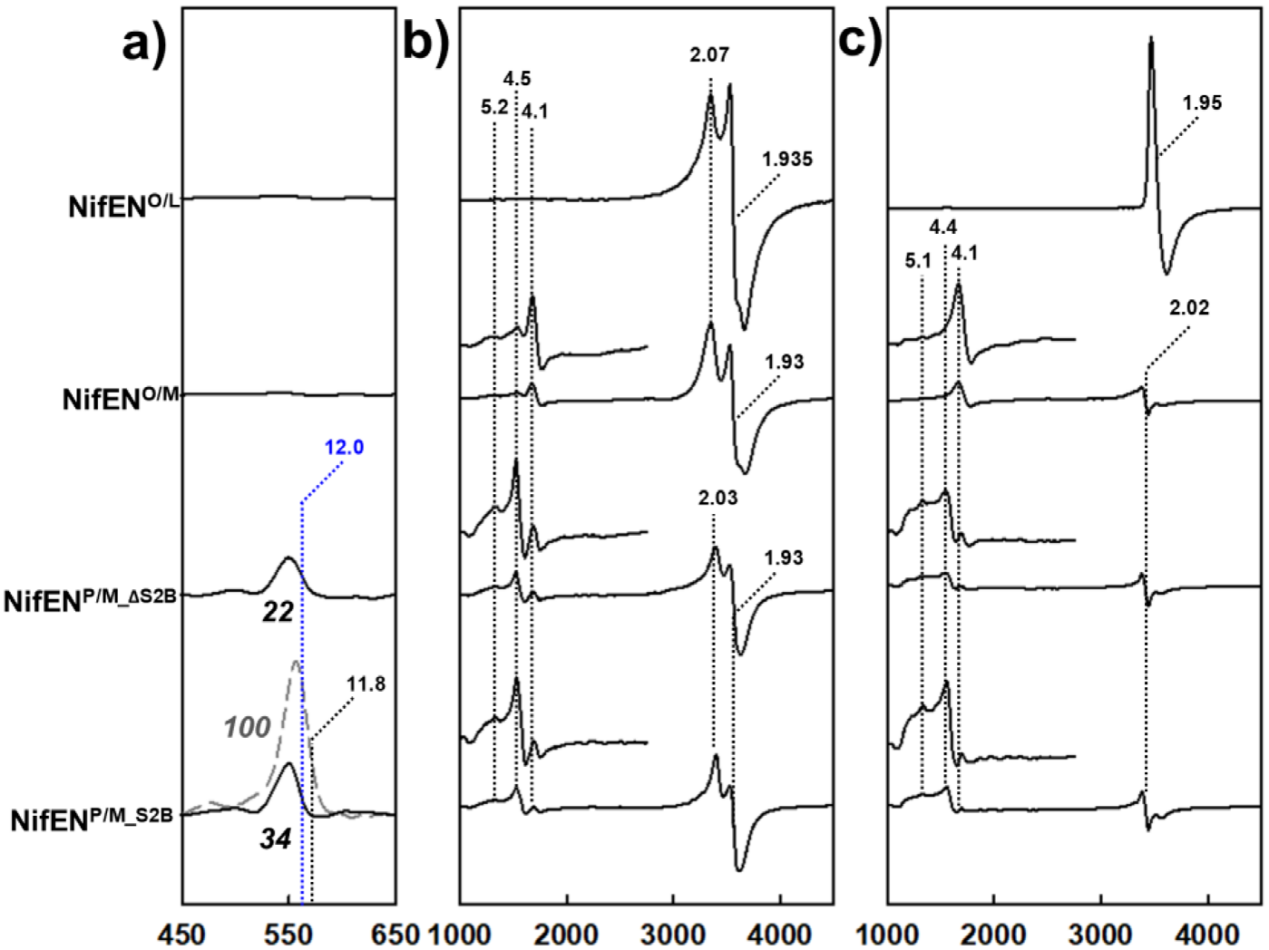
EPR properties of various NifEN proteins. (a) Parallel‐ and (b, c) perpendicular‐mode EPR spectra of NifEN^O/L^, NifEN^O/M^, NifEN^P/M_ΔS2B^, and NifEN^P/M_S2B^ in the IDS‐oxidized (a, c) and dithionite‐reduced (b) states. The *g* values are indicated. In a, the gray dashed line represents the P^OX^‐specific signal of NifDK at *g *= 11.8, compared with the P^OX^‐like signals of NifEN^P/M_ΔS2B^ and NifEN^P/M_S2B^ at *g*≈12. The intensity of the P^OX^‐specific signal in NifDK was set as 100%, and the relative intensities of the P^OX^‐like signals in NifEN^P/M_ΔS2B^ and NifEN^P/M_S2B^ were calculated accordingly. The partial P‐cluster occupancy (see (a) is consistent with the presence of immature P*‐clusters (comprising paired [Fe_4_S_4_] clusters) in NifEN^P/M_ΔS2B^ and NifEN^P/M_S2B^, which give rise to *S *= 1/2 signals with line shapes distinct from that of the O‐cluster‐associated *S *= 1/2 signal in NifEN^O/M^ (see b). In b, the feature at *g *= 4.1 is more pronounced than that at *g *= 4.5 for NifEN^O/M^, whereas the opposite trend is observed for NifEN^P/M_ΔS2B^ and NifEN^P/M_S2B^. Similarly, in c, the feature at *g *= 4.1 is more pronounced for NifEN^O/M^ than those for NifEN^P/M_ΔS2B^ and NifEN^P/M_S2B^, accompanied by a marked broadening of the features at *g* > 4.1.

Perpendicular‐mode EPR spectra of dithionite‐reduced samples further verified the active‐site cofactor composition (Figure 2b). As expected, NifEN^O/L^ exhibited a composite *S *= 1/2 EPR signal arising from the O‐ and L‐clusters and lacked features attributable to a mature M‐cluster [5]. In contrast, NifEN^O/M^ displayed the characteristic *S *= 3/2 signal of an NifEN‐bound M‐cluster [17], accompanied by a decrease in the *S *= 1/2 signal intensity, confirming success in the in vivo conversion of an L‐cluster to an M‐cluster. The two engineered variants, NifEN^P/M_ΔS2B^ and NifEN^P/M_S2B^, likewise exhibited mixed *S *= 1/2 and *S *= 3/2 EPR signatures. However, their spectra differed from that of NifEN^O/M^ in the line shapes of the M‐cluster‐associated *S *= 3/2 signals, particularly in the relative intensities of the features at *g *= 4.5 and 4.1. In addition, their *S *= 1/2 signals displayed line shapes distinct from the O‐cluster‐associated *S *= 1/2 signal of NifEN^O/M^. This observation is consistent with the partial P‐cluster occupancy of these variants (see Figure 2a) and supports the presence of immature, [Fe_4_S_4_]‐type P*‐cluster species.

Perpendicular‐mode EPR analysis of IDS‐oxidized samples provided additional evidence for the progressive reconstruction of the metal‐center architecture in the series of NifEN proteins (Figure 2c). The L‐cluster‐specific, *g *= 1.94 EPR signal observed for NifEN^O/L^ [17] was absent from all M‐cluster‐containing variants, whereas the engineered variants NifEN^P/M_ΔS2B^ and NifEN^P/M_S2B^ displayed broadened and more complex *S *= 3/2 features relative to that of NifEN^O/M^.

Together with the metal content analyses, the EPR data confirm successful installation of the intended cluster types and demonstrate that engineering of the electron‐transfer and active‐site environments measurably tunes the electronic structures of the target clusters. However, the spectroscopic differences relative to native NifDK indicate that the engineered metal clusters, particularly the M‐cluster, remain in environments that are not yet fully equivalent to those of the native enzyme.

### 2.3 Activity Assays

Consistent with the biochemically and spectroscopically verified reconstruction of metal‐center architecture, a stepwise recovery of in vitro activity was observed for the series of NifEN species as features of the NifDK electron‐transfer pathway and active‐site environment were progressively restored (Figure 3).

**FIGURE 3:**
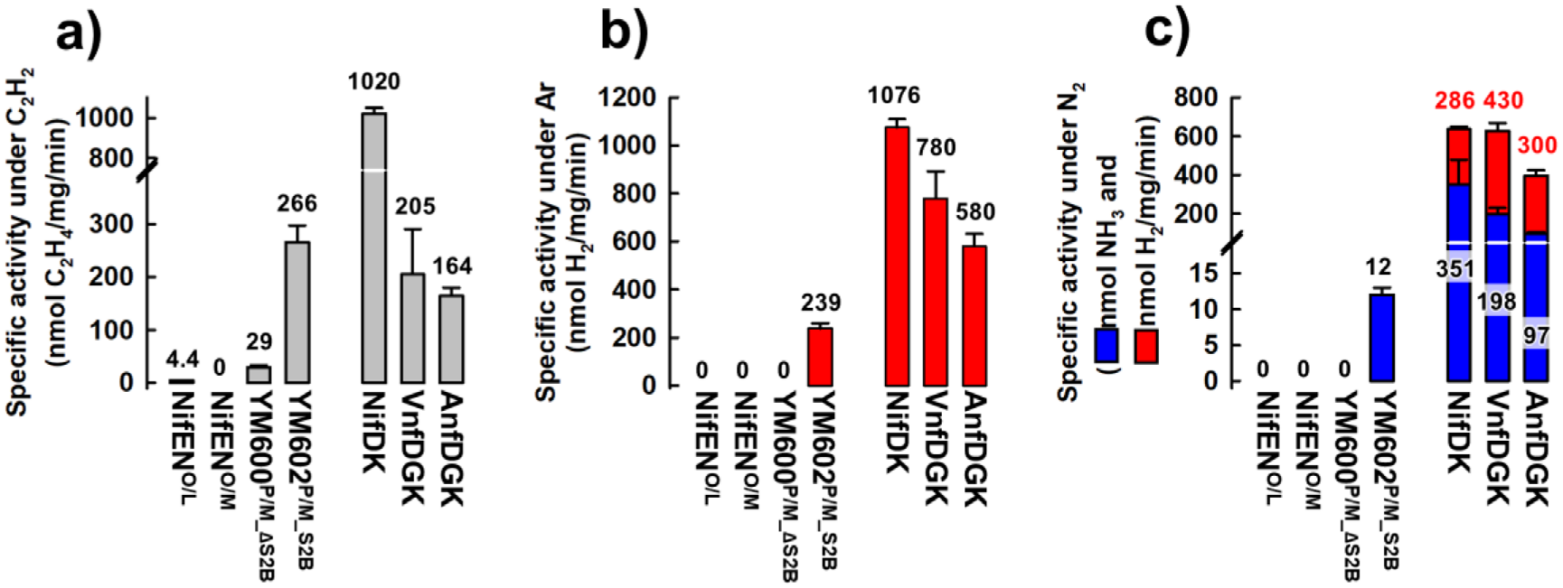
Activity analyses of various NifEN proteins. Specific activities of (a) C_2_H_2_‐reduction, (b) H^+^‐reduction, and (c) N_2_‐reduction by NifEN^O/L^, NifEN^O/M^, NifEN^P/M_ΔS2B^, and NifEN^P/M_S2B^, compared with those of NifDK, VnfDGK, and AnfDGK, in in vitro activity assays containing dithionite, MgATP and (i) NifH (for assays involving NifEN and NifDK), (ii) VnfH (for assays involving VnfDGK) or (iii) AnfH (for assays involving AnfDGK). All activities were normalized to the cofactor contents of the catalytic components of the respective nitrogenase systems. All data are presented as mean ± SD (*n *= 3), where *n* represents the number of experiments performed with independent protein preparations. The activity values for NifDK, VnfDGK, and AnfDGK were taken from literature [2, 3].

Acetylene reduction provided the first indication of functional recovery (Figure 3a). Under standard activity assay conditions for NifDK, NifEN^O/L^ displayed only marginal activity, whereas NifEN^O/M^ was inactive. In comparison, installation of both P‐ and M‐clusters resulted in increased catalytic performance. Of the two P/M‐cluster‐containing variants, NifEN^P/M_ΔS2B^ supported moderate acetylene reduction at approximately 3% of that observed for NifDK, demonstrating that restoration of the P→M electron‐transfer pathway alone can partially recover the ability to reduce easier substrates like C_2_H_2_. Further reconstruction of the belt‐sulfur (S2B) associated active‐site environment in NifEN^P/M_S2B^ increased activity by nearly an order of magnitude relative to NifEN^P/M_ΔS2B^, reaching approximately 26% of the activity of NifDK while exceeding those of the alternative V‐ and Fe‐only nitrogenases under the same conditions.

Proton reduction under Ar revealed a clear functional distinction of NifEN^P/M_S2B^ from the other examined NifEN species (Figure 3b). Neither NifEN^O/L^, NifEN^O/M^, nor NifEN^P/M_ΔS2B^ generated detectable H_2_, whereas NifEN^P/M_S2B^ exhibited robust proton‐reduction activity, reaching approximately 22%, 31%, and 41%, respectively, of the activities of NifDK, VnfDGK, and AnfDGK. Likewise, ATP‐dependent N_2_ reduction was also observed exclusively for NifEN^P/M_S2B^, which catalyzed NH_3_ formation at approximately 3.4%, 6.1%, and 12%, respectively, of the rates of NifDK, VnfDGK, and AnfDGK (Figure 3c). The ability of the engineered NifEN^P/M_S2B^ variant to pair with NifH and perform in vitro ATP‐dependent ^15^N_2_ reduction was further verified by the appearance of the ^15^NH_4_^+^‐specific doublet at 6.97 and 7.15 ppm in the frequency‐selective ^1^H pulse NMR spectrum (Figure S7).

The acquisition of both H_2_‐evolution and N_2_‐reduction activities by NifEN^P/M_S2B^ represents a significant gain of function, as these two activities are considered mechanistically coupled in nitrogenase catalysis [2, 3, 4, 5, 6, 7, 8]. At the same time, NifEN^P/M_S2B^ remains substantially less active than NifDK in N_2_ reduction and exhibits no detectable H_2_ evolution under N_2_, indicating that some key features of the native catalytic machinery remain incompletely restored.

### 2.4 NanoSIMS Analysis

To determine whether the in vitro N_2_ reduction activity of NifEN^P/M_S2B^ could be translated into biological nitrogen fixation, *E. coli* strains expressing NifEN^P/M_S2B^, NifEN^P/M_ΔS2B^, and NifEN^O/M^ were cultivated under ^1^ ^5^N_2_ and subsequently analyzed for ^15^N assimilation by nanoscale secondary ion mass spectrometry (nanoSIMS) (Figure 4). Using a previously established protocol [38, 39, 40], cells were grown under nitrogen‐limited conditions and allowed to transition from NH_4_^+^‐supported growth to dependence on atmospheric ^15^N_2_, enabling direct assessment of nitrogen fixation through incorporation of isotopically labeled nitrogen into cellular biomass.

**FIGURE 4:**
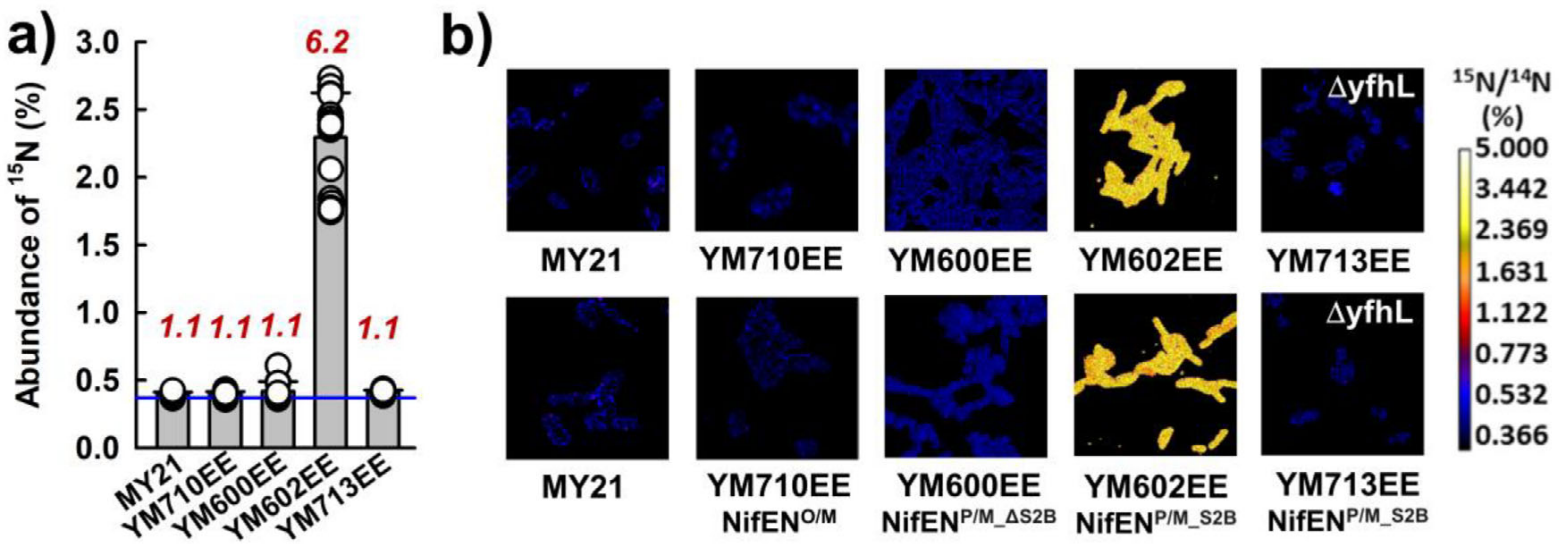
Diazotrophic nitrogen assimilation by *E. coli* expressing NifEN^P/M_S2B^. (a) Statistical analysis of secondary ion images derived from (b) nanoSIMS experiments of five *E. coli* strains grown under 100% ^15^N_2_: MY21, a nitrogenase‐free, *yfhL*‐replete strain; YM710EE, a NifEN^O/M^‐expressing, *yfhL*‐replete strain; YM600EE, a NifEN^P/M_ΔS2B^‐expressing, *yfhL*‐replete strain; YM602EE, a NifEN^P/M_S2B^‐expressing, *yfhL*‐replete strain; and YM713EE, a NifEN^P/M_S2B^‐expressing, *yfhL*‐deficient strain. The ^15^N abundance of each strain was calculated based on data collected in 6 different regions of interest (ROI) from 2–4 independent samples. Two representative nanoSIMS images of these samples are shown in the figure. The average values of ^15^N abundance (^15^N/^14^N) are: MY21 (*n *= 2), 0.40% ± 0.02%; YM710EE (*n *= 4), 0.40% ± 0.02%; YM600EE (*n *= 2), 0.42% ± 0.08%; YM602EE (*n *= 4), 2.29% ± 0.33%; YM713EE (*n *= 3), 0.42% ± 0.01%. In a, data are expressed as mean ± SD, and individual data points are indicated as open circles. Given the natural ^15^N abundance (^15^N/^14^N) of 0.37% (indicated by a horizontal blue line in panel a), the ^15^N enrichment level is 6.2‐fold for YM602EE.

Notably, the in vivo nanoSIMS profiles of the various NifEN species closely mirrored their in vitro activity patterns (Figure 4). Similar to the nitrogenase‐free control (strain MY21), cells expressing NifEN^O/M^ (strain YM710EE) showed negligible ^15^N enrichment above natural abundance, indicating the absence of physiologically relevant N_2_ reduction. Likewise, cells expressing NifEN^P/M_ΔS2B^ (strain YM600EE) exhibited only minimal ^15^N incorporation, consistent with their inability to support NH_3_ formation in vitro. In contrast, expression of NifEN^P/M_S2B^ resulted in pronounced ^15^N enrichment throughout the cell population (strain YM602EE), corresponding to an average ^15^N/^14^N ratio approximately 6.2‐fold above natural abundance. Interestingly, deletion of the endogenous ferredoxin gene *yfhL* in this background (strain YM713EE) abolished ^15^N incorporation, pointing to YfhL—a two [Fe_4_S_4_] cluster‐containing, low‐potential ferredoxin—as a likely physiological electron donor for the engineered NifH/NifEN^P/M_S2B^ system.

### 2.5 Structural Prediction

To identify structural features that may still limit catalytic performance, the most active engineered variant, NifEN^P/M_S2B^, was analyzed using Boltz‐2.2.1 [43, 44]. The predicted structure modeled two regions that remained incompletely reconstructed relative to NifDK: the P‐cluster ligation environment and the region surrounding the belt‐sulfur sites of the M‐cluster (Figure 5).

**FIGURE 5:**
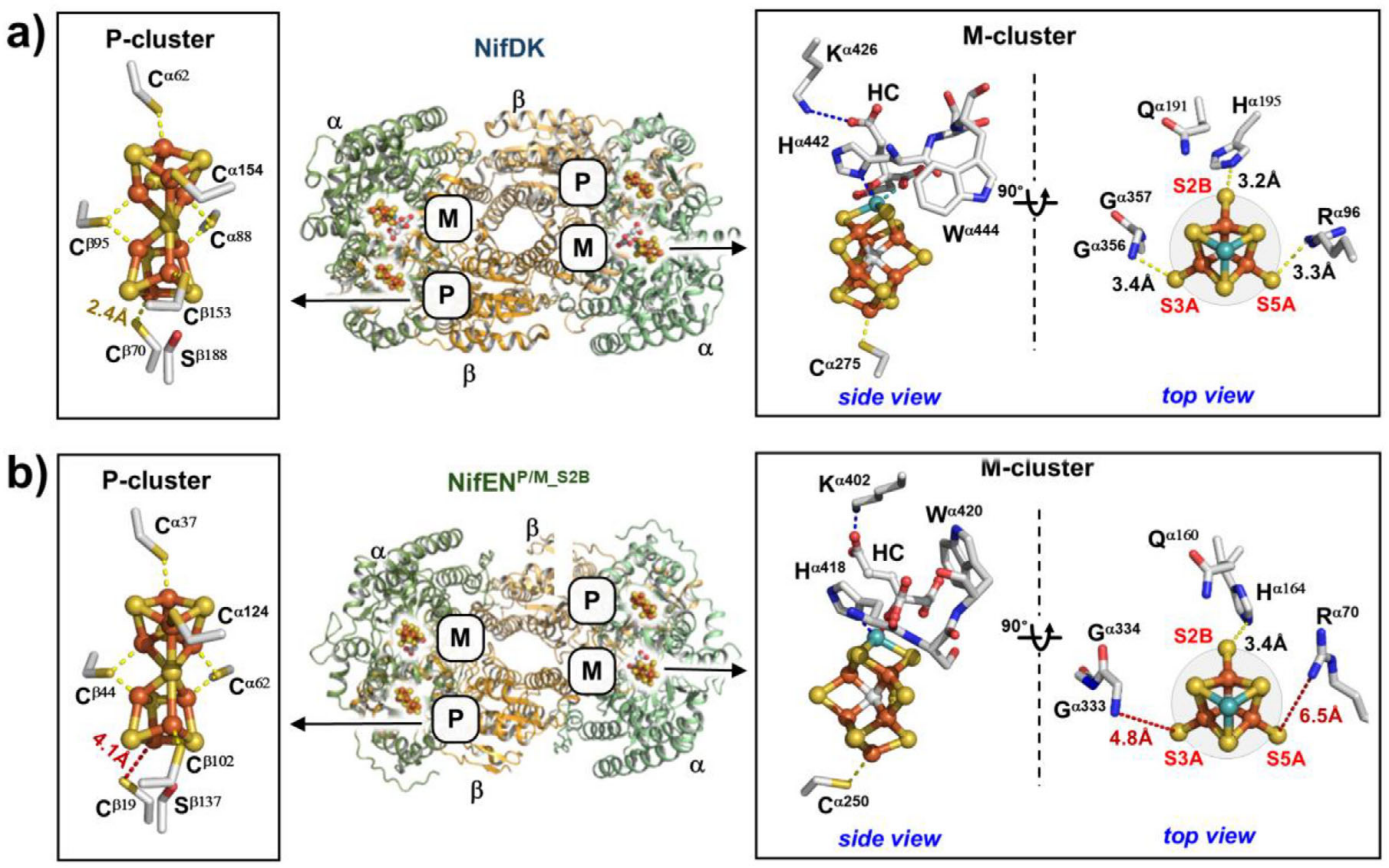
The P‐ and M‐cluster regions in NifDK and NifEN^P/M_S2B^. (a) X‐ray crystal structure of NifDK. Overview of the α_2_​β_2_​‐heterotetrameric NifDK, showing the relative positions of the P‐ and M‐clusters (middle). Detailed view of the native [Fe_8_​S_7_​] P‐cluster environment, coordinated by six cysteine ligands and one serine residue (left). The active‐site environment surrounding the M‐cluster features terminal ligands (C^α275^, H^α442^), the homocitrate anchor (K^α426^), and the “switch/lock” (H^α442^ /W^α444^) (right, side view). Productive catalysis is supported by the precisely organized belt‐sulfur region, including the key residues at the S3A site (G*^α^* ^357^), the S2B site (Q*^α^* ^191^, H^α195^), and the S5A site (R^α96^) (right, top view). The G*^α^* ^357^—S3A, H^α195^—S2B and R^α96^—S5A distances are 3.4, 3.2, and 3.3 Å, respectively. (b) Boltz‐2.2.1 structural prediction of the engineered NifEN^P/M_S2B^ variant. Overview of the α_2_​β_2_​‐heterotetrameric NifEN^P/M_S2B^, showing the relative positions of the P‐ and M‐clusters (middle). The engineered P‐cluster ligation environment remains incompletely reconstructed, as the C^β19^ ligand is positioned 4.1 Å from the cluster, farther than the corresponding C^β70^ ligand in NifDK (left). Structural discrepancies of the active‐site environment surrounding the M‐cluster (right). Although proton gating is restored at the S2B site (Q^α160^, H^α164^), the native R^α70^—retained in NifEN^P/M_S2B^ and expected to mimic R^α96^ in NifDK based on amino acid sequence alignment (*see* Figure S3)—is predicted to occupy a location structurally resembling R^α97^ in NifDK (see Figure S9). Furthermore, the increased distances between the protein backbone and the belt sites (right, top view)—4.8 Å for S3A and 6.5 Å for S5A (dark red dashed lines)—indicate an incompletely organized belt region in NifEN^P/M_S2B^, likely accounting for the low catalytic deficiency of this engineered variant. The metal centers are depicted and colored as described in Figure 1. PyMOL was used to generate this figure, using PDB entry 3U7Q [11] for NifDK, and Boltz‐2.2.1 prediction for NifEN^P/M_S2B^.

With respect to the first divergence at the P‐cluster site, despite the successful installation of a P‐cluster like structure in NifEN^P/M_S2B^, the engineered C^β19^ ligand is predicted to remain positioned 4.1 Å from one Fe atom of the P‐cluster (Figure 5b), substantially farther than its counterpart in NifDK (Figure 5a). This prediction suggests that the electronic properties and redox flexibility of the reconstructed P‐cluster may not yet fully reproduce those of the native electron‐transfer center, consistent with the reduced catalytic efficiency of the engineered protein.

A second discrepancy is predicted to reside in the belt‐sulfur region of the M‐cluster (Figure 5b). In NifEN^P/M_S2B^, the protein backbone is modeled at a position substantially farther from two belt sulfurs, with G^α333^ located 4.8 Å from S3A and R^α70^ located 6.5 Å from S5A, compared with the corresponding G^356^—S3A and R^α96^—S5A distances observed in NifDK. These presumably increased separations likely contribute to the reduced catalytic efficiency, particularly in light of our previously proposed model involving sequential binding and reduction of N_2_ at the S3A, S2B, and S5A sites (Figure S8) [34]. Interestingly, although sequence alignment places R^α70^ of NifEN^P/M_S2B^ at the position corresponding to R^α96^ in NifDK (Figure S3), structural modeling indicates that its predicted location more closely resembles that of R^α97^ in NifDK (Figure S9). The potential presence of an R^α97^‐equivalent residue in NifEN^P/M_S2B^, which interacts with the homocitrate ligand and helps organize the local hydrogen‐bonding network, may partially account for its ability to evolve H_2_. However, the predicted absence of an R^α96^‐equivalent residue likely prevents NifEN^P/M_S2B^ from recapitulating the structural role of this critical residue in defining the catalytic environment around S5A, thereby limiting the efficiency of N_2_ reduction.

## 3 Discussion

The progressive engineering of NifEN in a clean heterologous expression background establishes a direct relationship between reconstruction of the metal‐center architecture and recovery of nitrogenase function. Installation of the mature M‐cluster alone was insufficient to support catalysis, as NifEN^O/M^ remained inactive despite successful cofactor maturation. Likewise, restoration of the P→M electron‐transfer pathway alone yielded only partial activity, with NifEN^P/M_ΔS2B^ able to perform moderate C_2_H_2_ reduction but unable to reduce H^+^ or N_2_. ATP‐dependent H^+^‐ and N_2_‐reduction emerged only after reconstruction of both the electron‐transfer relay and the S2B‐associated active‐site environment in NifEN^P/M_S2B^, demonstrating that productive catalysis requires coordinated optimization of these structural features.

Restoration of N_2_ reduction in NifEN^P/M_S2B^ is particularly informative because it highlights the essential role of the S2B site in nitrogenase catalysis. Conversely, the substantially lower activity of NifEN^P/M_S2B^ relative to native nitrogenases—attributed in large part, according to structural modeling, to the incompletely reconstructed S3A and S5A regions—illustrates the importance of functional differentiation within the cofactor belt region. Together, these findings indicate that productive N_2_ reduction requires an intact belt region with the precise structural organization of the individual belt sites (S3A, S2B, and S5A). Along with the non‐native P‐cluster ligation geometry, the incomplete reconstruction of the cofactor belt region likely accounts for the remaining catalytic deficiency while also identifying clear targets for further engineering toward a fully nitrogenase‐like catalyst.

Beyond its mechanistic implications, construction of engineered NifEN variants like NifEN^P/M_S2B^ also simplifies the challenge of heterologous nitrogenase expression. Unlike the native system, which requires maturation of the M‐cluster on NifEN and transfer of the mature cofactor to NifDK, NifEN^P/M_S2B^ combines self‐assembly of the M‐cluster with ATP‐dependent N_2_ reduction in a single scaffold. This combination eliminates the need to express NifDK and bypasses the otherwise essential M‐cluster transfer step, thereby considerably reducing the genetic and biosynthetic complexity required to establish a functional nitrogen‐fixing system in heterologous hosts.

## 4 Conclusions

In summary, this work establishes NifEN as a catalytically competent nitrogenase mimic capable of self‐assembling a mature cofactor, rather than receiving a preassembled cofactor from another biosynthetic protein, through systematic reconstruction of structural features governing electron transfer and active‐site catalysis. The engineered NifEN^P/M_S2B^ variant supports ATP‐dependent N_2_ reduction both in vitro and in vivo, providing a streamlined platform for mechanistic studies while substantially simplifying heterologous nitrogenase expression. Together, these findings advance the understanding of the structural basis of biological nitrogen fixation and establish a foundation for engineering increasingly efficient biological and bioinspired nitrogen‐fixing systems.

## Author Contributions

**Yimo Yang**: investigation, validation, visualization, formal analysis. **Chi Chung Lee**: investigation, formal analysis, validation, visualization. **Steve Ortiz**: investigation. **Markus W. Ribbe**: conceptualization, funding acquisition, writing – original draft, writing – review and editing, visualization, validation, formal analysis, project administration, supervision, data curation, resources, investigation. **Yilin Hu**: conceptualization, investigation, funding acquisition, writing – original draft, validation, visualization, writing – review and editing, formal analysis, project administration, data curation, supervision, resources.

## Conflicts of Interest

The authors declare no conflicts of interest.

## Supporting information




**Supporting File**: anie74912‐sup‐0001‐SuppMat.pdf.

## Data Availability

The data that support the findings of this study are available from the corresponding author upon reasonable request.

## References

[1] A. Lago‐Maciel , M. Herzog , and J. G. Rebelein , “Functional Insights Into the Nitrogenase‐Like Enzyme Superfamily,” Trends in Biochemical Sciences 51 (2026): 187–202, 10.1016/j.tibs.2025.11.009. 41483962

[2] Y. Hu , C. C. Lee , M. Grosch , J. B. Solomon , W. Weigand , and M. W. Ribbe , “Enzymatic Fischer–Tropsch‐Type Reactions,” Chemical Reviews 123 (2023): 5755–5797, 10.1021/acs.chemrev.2c00612. PMC10476197 36542730

[3] A. J. Jasniewski , C. C. Lee , M. W. Ribbe , and Y. Hu , “Reactivity, Mechanism, and Assembly of the Alternative Nitrogenases,” Chemical Reviews 120 (2020): 5107–5157, 10.1021/acs.chemrev.9b00704. PMC7491575 32129988

[4] R. R. Eady , “Structure−Function Relationships of Alternative Nitrogenases,” Chemical Reviews 96 (1996): 3013–3030, 10.1021/cr950057h. 11848850

[5] B. K. Burgess and D. J. Lowe , “Mechanism of Molybdenum Nitrogenase,” Chemical Reviews 96 (1996): 2983–3012, 10.1021/cr950055x. 11848849

[6] D. C. Rees , A. F. Tezcan , C. A. Haynes , et al., “Structural Basis of Biological Nitrogen Fixation,” Philosophical Transactions of the Royal Society A: Mathematical, Physical and Engineering Sciences 363 (2005): 971. 10.1098/rsta.2004.1539 15901546

[7] H. L. Rutledge and F. A. Tezcan , “Electron Transfer in Nitrogenase,” Chemical Reviews 120 (2020): 5158–5193, 10.1021/acs.chemrev.9b00663. PMC7466952 31999100

[8] S. T. Stripp , B. R. Duffus , V. Fourmond , et al., “Second and Outer Coordination Sphere Effects in Nitrogenase, Hydrogenase, Formate Dehydrogenase, and CO Dehydrogenase,” Chemical Reviews 122 (2022): 11900–11973, 10.1021/acs.chemrev.1c00914. PMC9549741 35849738

[9] H. Schindelin , C. Kisker , J. L. Schlessman , J. B. Howard , and D. C. Rees , “Structure of ADP·AIF_4_–‐Stabilized Nitrogenase Complex and Its Implications for Signal Transduction,” Nature 387 (1997): 370–376, 10.1038/387370a0. 9163420

[10] H. L. Rutledge , B. D. Cook , H. P. M. Nguyen , M. A. Herzik , and F. A. Tezcan , “Structures of the Nitrogenase Complex Prepared Under Catalytic Turnover Conditions,” Science 377 (2022): 865–869, 10.1126/science.abq7641. PMC9949965 35901182

[11] T. Spatzal , M. Aksoyoglu , L. Zhang , et al., “Evidence for Interstitial Carbon in Nitrogenase FeMo Cofactor,” Science 334 (2011): 940, 10.1126/science.1214025. PMC3268367 22096190

[12] K. M. Lancaster , M. Roemelt , P. Ettenhuber , et al., “X‐ray Emission Spectroscopy Evidences a Central Carbon in the Nitrogenase Iron‐Molybdenum Cofactor,” Science 334 (2011): 974–977, 10.1126/science.1206445. PMC3800678 22096198

[13] J. A. Wiig , Y. Hu , C. C. Lee , and M. W. Ribbe , “Radical SAM‐Dependent Carbon Insertion Into the Nitrogenase M‐Cluster,” Science 337 (2012): 1672–1675, 10.1126/science.1224603. PMC3836454 23019652

[14] J. T. Kaiser , Y. Hu , J. A. Wiig , D. C. Rees , and M. W. Ribbe , “Structure of Precursor‐Bound NifEN: A Nitrogenase FeMo Cofactor Maturase/Insertase,” Science 331 (2011): 91–94, 10.1126/science.1196954. PMC3138709 21212358

[15] A. W. Fay , M. A. Blank , C. C. Lee , et al., “Spectroscopic Characterization of the Isolated Iron–Molybdenum Cofactor (FeMoco) Precursor From the Protein NifEN,” Angewandte Chemie International Edition 50 (2011): 7787–7790, 10.1002/anie.201102724. PMC3395727 21726031

[16] M. C. Corbett , Y. Hu , A. W. Fay , M. W. Ribbe , B. Hedman , and K. O. Hodgson , “Structural Insights Into a Protein‐Bound Iron‐Molybdenum Cofactor Precursor,” PNAS 103 (2006): 1238–1243, 10.1073/pnas.0507853103. PMC1360540 16423898

[17] J. B. Solomon , C. C. Lee , A. J. Jasniewski , M. F. Rasekh , M. W. Ribbe , and Y. Hu , “Heterologous Expression and Engineering of the Nitrogenase Cofactor Biosynthesis Scaffold NifEN,” Angewandte Chemie International Edition 59 (2020): 6887–6893, 10.1002/anie.201916598. 32022452

[18] C. C. Lee , K. Górecki , M. Stang , M. W. Ribbe , and Y. Hu , “Cofactor Maturase NifEN: A Prototype Ancient Nitrogenase?,” Science Advances 10 (2024): eado6169, 10.1126/sciadv.ado6169. PMC11168457 38865457

[19] A. W. Fay , M. A. Blank , J. G. Rebelein , et al., “Assembly Scaffold NifEN: A Structural and Functional Homolog of the Nitrogenase Catalytic Component,” PNAS 113 (2016): 9504–9508, 10.1073/pnas.1609574113. PMC5003292 27506795

[20] Y. Hu , A. W. Fay , C. C. Lee , J. A. Wiig , and M. W. Ribbe , “Dual Functions of NifEN: Insights Into the Evolution and Mechanism of Nitrogenase,” Dalton Transactions 39 (2010): 2964, 10.1039/b922555b. PMC3030252 20221527

[21] Y. Hu and M. W. Ribbe , “Decoding the Nitrogenase Mechanism: The Homologue Approach,” Accounts of Chemical Research 43 (2010): 475–484, 10.1021/ar900254x. PMC2840065 20030377

[22] J. M. Yoshizawa , A. W. Fay , C. C. Lee , Y. Hu , and M. W. Ribbe , “Insertion of Heterometals Into the NifEN‐Associated Iron–Molybdenum Cofactor Precursor,” Journal of Biological Inorganic Chemistry 15 (2010): 421–428, 10.1007/s00775-009-0614-5. PMC2830607 19967421

[23] Y. Hu , J. M. Yoshizawa , A. W. Fay , C. C. Lee , J. A. Wiig , and M. W. Ribbe , “Catalytic Activities of NifEN: Implications for Nitrogenase Evolution and Mechanism,” PNAS 106 (2009): 16962–16966, 10.1073/pnas.0907872106. PMC2761346 19805110

[24] Y. Hu , M. C. Corbett , A. W. Fay , et al., “FeMo Cofactor Maturation on NifEN,” PNAS 103 (2006): 17119–17124, 10.1073/pnas.0602647103. PMC1859895 17050696

[25] B. Neumann , K. A. Brandon , R. Quechol , et al., “Structural Insights Into Metallocluster Trafficking in the Nitrogenase Assembly Scaffold NifEN,” Nature Catalysis 9 (2026): 281–294, 10.1038/s41929-026-01489-9. PMC13031127 41908675

[26] C. C. Lee , K. Góreck , M. Stang , and M. W. Ribbe , “Cofactor Maturase NifEN: A Prototype Ancient Nitrogenase?,” Science Advances 10 (2024): eado6169, 10.1126/sciadv.ado6169. PMC11168457 38865457

[27] H. Thomann , M. Bernardo , W. E. Newton , and D. R. Dean , “N Coordination of FeMo Cofactor Requires His‐195 of the MoFe Protein Alpha Subunit and Is Essential for Biological Nitrogen Fixation,” PNAS 88 (1991): 6620–6623, 10.1073/pnas.88.15.6620. PMC52139 11607203

[28] M. J. Dilworth , K. Fisher , C. H. Kim , and W. E. Newton , “Effects on Substrate Reduction of Substitution of Histidine‐195 by Glutamine in the α‐Subunit of the MoFe Protein of Azotobactervinelandii Nitrogenase,” Biochemistry 37 (1998): 17495–17505, 10.1021/bi9812017. 9860864

[29] K. Fisher , M. J. Dilworth , C. H. Kim , and W. E. Newton , “ *Azotobacter vinelandii* Nitrogenases Containing Altered MoFe Proteins With Substitutions in the FeMo‐Cofactor Environment: Effects on the Catalyzed Reduction of Acetylene and Ethylene,” Biochemistry 39 (2000): 2970–2979, 10.1021/bi992092e. 10715117

[30] M. C. Durrant , A. Francis , D. J. Lowe , W. E. Newton , and K. Fisher , “Evidence for a Dynamic Role for Homocitrate During Nitrogen Fixation: The Effect of Substitution at the α‐Lys426 Position in MoFe‐protein of *Azotobacter vinelandii* ,” Biochemical Journal 397 (2006): 261–270, 10.1042/BJ20060102. PMC1513279 16566750

[31] K. Fisher , H. D. Hare , and W. E. Newton , “Another Role for CO With Nitrogenase? CO Stimulates Hydrogen Evolution Catalyzed by Variant *Azotobacter Vinelandii* Mo‐Nitrogenases,” Biochemistry 53 (2014): 6151–6160, 10.1021/bi500546k. 25203280

[32] Z. Maskos , K. Fisher , M. Sørlie , W. E. Newton , and B. J. Hales , “Variant MoFe Proteins of *Azotobacter vinelandii*: Effects of Carbon Monoxide on Electron Paramagnetic Resonance Spectra Generated during Enzyme Turnover,” Journal of Biological Inorganic Chemistry 10 (2005): 394–406, 10.1007/s00775-005-0648-2. 15887041

[33] B. Schmid , M. W. Ribbe , O. Einsle , et al., “Structure of a Cofactor‐Deficient Nitrogenase MoFe Protein,” Science 296 (2002): 352. 10.1126/science.1070010 11951047

[34] W. Kang , C. C. Lee , A. J. Jasniewski , M. W. Ribbe , and Y. Hu , “Structural Evidence for a Dynamic Metallocofactor during N_2_ Reduction by Mo‐Nitrogenase,” Science 368 (2020): 1381–1385, 10.1126/science.aaz6748. PMC8410457 32554596

[35] Y. Hu , M. C. Corbett , A. W. Fay , et al., “Nitrogenase Fe Protein: A Molybdate/Homocitrate Insertase,” PNAS 103 (2006): 17125–17130, 10.1073/pnas.0602651103. PMC1859896 17062756

[36] J. M. Yoshizawa , M. A. Blank , A. W. Fay , et al., “Optimization of FeMoco Maturation on NifEN,” Journal of the American Chemical Society 131 (2009): 9321–9325, 10.1021/ja9035225. PMC2716083 19514721

[37] Y. Hu , A. W. Fay , B. Schmid , B. Makar , and M. W. Ribbe , “Molecular Insights Into Nitrogenase FeMoco Insertion: TRP‐444 of MoFe Protein Alpha‐Subunit Locks FeMoco in its Binding Site,” Journal of Biological Chemistry 281 (2006): 30534–30541, 10.1074/jbc.M605527200. 16893897

[38] Y. A. Liu , C. C. Lee , K. Górecki , et al., “Heterologous Synthesis of a Simplified Nitrogenase Analog in *Escherichia coli* ,” Science Advances 11 (2025): eadw6785, 10.1126/sciadv.adw6785. PMC12047441 40315313

[39] J. B. Solomon , C. C. Lee , Y. A. Liu , C. Duffin , M. W. Ribbe , and Y. Hu , “Ammonia Synthesis via an Engineered Nitrogenase Assembly Pathway in *Escherichia coli* ,” Nature Catalysis 7 (2024): 1130–1141, 10.1038/s41929-024-01229-x. PMC11661828 39713742

[40] R. Quechol , Y. Yang , C. C. Lee , M. W. Ribbe , and Y. Hu , “Minimal ATP‐Independent N_2_‐Reducing Systems Defined by L‐Cluster‐Bound Nitrogenase Assembly Platforms,” Angewandte Chemie International Edition 65 (2026): e2968123, 10.1002/anie.2968123. PMC13245594 42046444

[41] L. Zheng and R. H. White , “Purification of the *Azotobacter vinelandii* nifV‐Encoded Homocitrate Synthase,” Journal of Bacteriology 179 (1997): 5963–5966, 10.1128/jb.179.18.5963-5966.1997. PMC179493 9294461

[42] A. J. Pierik , H. Wassink , H. Haaker , and W. R. Hagen , “Redox Properties and EPR Spectroscopy of the P Clusters of *Azotobacter Vinelandii* MoFe Protein,” European Journal of Biochemistry 212 (1993): 51–61, 10.1111/j.1432-1033.1993.tb17632.x. 8383042

[43] S. Passaro , G. Corso , J. Wohlwend , et al., “Boltz‐2: Towards Accurate and Efficient Binding Affinity Prediction,” bioRxiv (2025), preprint, 10.1101/2025.06.14.659707.

[44] J. Wohlwend , G. Corso , S. Passaro , et al., “Boltz‐1 Democratizing Biomolecular Interaction Modeling,” bioRxiv (2025), preprint, 10.1101/2024.11.19.624167.

