# Supplementary material for: Gain‐of‐Function Engineering of the NifEN Scaffold Into a Self‐Assembling Nitrogenase
Source: Angew Chem Int Ed Engl. 2026 Sep 21;65(45):e8683437. doi: 10.1002/anie.8683437 (PMC13725552; doi:10.1002/anie.8683437)
Supplement: Supplementary file 1 [file ANIE-65-e8683437-s001.pdf]

Supporting Information

Gain-of-Function Engineering of the NifEN Scaffold into a Self-Assembling Nitrogenase

Yimo Yang,+[a] Chi Chung Lee,+[a], Steve Ortiz,[a] Markus W. Ribbe,*[a][b] Yilin Hu*[a]

[a]Department of Molecular Biology and Biochemistry, University of California, Irvine, CA 92697-3900
[b]Department of Chemistry, University of California, Irvine, CA 92697-2025

*Correspondence should be addressed to mribbe@uci.edu and yilinh@uci.edu.
+These authors contributed equally to this work.

Experimental Section

General procedure

Unless specified otherwise, all chemicals were purchased from Sigma-Aldrich (St. Louis, MO) and Thermo Fisher Scientific (Waltham, MA). All experiments were conducted in a glove box or on a Schlenk line under an Ar atmosphere, with an O_2 concentration of <3 ppm.

Strain construction

Previously, *nifH*, *nifM*, *nifE* (with a polyhistidine tag), and *nifN* from *A. vinelandii*, together with *nifS3*, *nifU3*, and *nifB* from *M. acetivorans*, were synthesized and cloned into pRSFDuet-1 and pCDFDuet-1 (GenScript, Piscataway, NJ) and co-expressed in *E. coli* strain MY21, a BL21(DE3)-derived Δ*iscR* strain. The resulting strain (YM578EE) supports the formation of (i) a $[Fe_4S_4]$-bound NifH (via *nifH* and *nifM*), (ii) an L-cluster–bound NifB (via *nifS3*, *nifU3*, and *nifB*), and (iii) an O-cluster–replete but L-cluster–deficient apo-NifEN (via *nifE* and *nifN*). Subsequent *in vivo* transfer of the L-cluster from NifB to apo-NifEN yields NifEN containing both O- and L-clusters (designated $NifEN^{O/L}$). Taken together, this system (YM578EE) enabled heterologous synthesis of a two-component nitrogenase analog comprising non-tagged NifH and His-tagged $NifEN^{O/L}$.[1]

Using a similar strategy, *nifH*, *nifM*, *nifV*, *nifE* (with a polyhistidine tag), and *nifN* from *A. vinelandii*, along with *nifS3*, *nifU3*, and *nifB* from *M. acetivorans*, were synthesized and cloned into pRSFDuet-1 and pCDFDuet-1 (GenScript, Piscataway, NJ) and co-expressed in *E. coli* strain MY21. The resulting strain (YM710EE, *see* Table S1 and Figure S4) supports formation of $NifEN^{O/L}$ as described above, followed by *in vivo* L-to-M cluster conversion via NifH-mediated insertion of Mo (supplied in the medium) and homocitrate (synthesized by NifV), yielding NifEN containing both O- and M-clusters (designated $NifEN^{O/M}$).

Building on this system, *nifH*, *nifM*, *nifZ*, *nifV*, *nifE** (with a polyhistidine tag), and *nifN** from *A. vinelandii*, together with *nifS3*, *nifU3*, and *nifB* from *M. acetivorans*, were synthesized and cloned into pRSFDuet-1 and pCDFDuet-1 (GenScript, Piscataway, NJ) and co-expressed in *E. coli* strain MY21. The NifE*N* variant incorporates the "missing" ligands required to accommodate a P-cluster while restoring the second terminal ligand, the homocitrate anchor, and the "switch/lock" residues required for M-cluster coordination and stabilization (*see* Table S1 and Figure S4 for details). These modifications support the formation of a P*-cluster (a precursor consisting of a pair of $[Fe_4S_4]$-like clusters) and an L-cluster–containing NifEN. Subsequent *in vivo* P*-to-P and L-to-M conversions—both mediated by NifH—yield NifEN containing both P- and M-clusters.

Within the NifE* sequence, belt-site residues were either kept as-is in the modified strain above to maintain deficiency at the S2B site (ΔS2B), or further modified through gene synthesis (GenScript, Piscataway, NJ) to restore the S2B site (*see* Table S1 and Figure S4 for details). These designs enabled the production of two P- and M-cluster containing variants with defined belt-site configurations: (i) S2B-site-deficient (designated $NifEN^{P/M\_\Delta S2B}$; strain YM600EE) and (ii) S2B-site-replete (designated $NifEN^{P/M\_S2B}$; strain YM602EE). The S2B-site-replete $NifEN^{P/M\_S2B}$ variant was also expressed in MY25,[1] a derivative of MY21[2] carrying a deletion of *yfhL*, the gene encoding a low-potential, two-$[Fe_4S_4]$-cluster ferredoxin (*see* Table S1 for details). This approach generated an *E. coli* strain that, in the absence of YfhL, expressed two-component nitrogenase analogs comprising non-tagged NifH and His-tagged NifEN variant containing both P- and M-clusters and an S2B–replete cofactor environment (strain YM713EE).

Cell growth and protein purification

E. coli strains YM578EE, YM600EE, YM602EE, and YM710EE were grown in 10-L batches in LB medium (Difco) supplemented with 50 mM MOPS/NaOH (pH 7.4), 25 mM glucose, 2 mM ferric ammonium citrate, kanamycin (30 mg/L), and streptomycin (30 mg/L) in a BIOFLO 415 fermenter (New Brunswick Scientific) at 37°C with agitation at 200 rpm and airflow of 10 liters/min. When the optical density at 600 nm (OD_{600}) reached 0.5, the airflow was terminated, and the fermenter was purged with N_2 (ultrahigh purity) at a rate of 1.5 L/min; additionally, the temperature was lowered to 24°C. Once the temperature reached 24°C, 2 mM cysteine was added, and the expression of nitrogenase proteins was induced by the addition of 250 µM IPTG. Protein expression was allowed to continue for 16 h, followed by harvesting of the cells by centrifugation using a Thermo Fisher Scientific Legend XTR centrifuge. The heterologously expressed, His-tagged NifEN proteins were purified from the *E. coli* expression strains by IMAC using a method adapted from the purification of the His-tagged nitrogenase proteins from *A. vinelandii*.[1-4]

A. vinelandii strains DJ1162 and DJ1141 expressing His-tagged NifH and NifDK,[3,5] respectively, were grown in 180-L batches in Burke's minimal medium supplemented with 2 mM ammonium acetate in a 200-L fermenter (New Brunswick Scientific) at 30°C with agitation at 100 rpm and airflow of 30 L/min. Cell growth was monitored at 436 nm (OD_{436}) using a Spectronic 20 Genesys spectrometer (Spectronic Instruments) and, upon depletion of ammonia, cells were derepressed for 3 h before harvesting by a flow-through centrifugal harvester (Cepa). Published methods were used to purify His-tagged NifH and NifDK from *A. vinelandii* strains.[3,5]

Metal analysis

The metal contents of NifEN proteins were determined by inductively coupled plasma optical emission spectroscopy (ICP-OES) using the iCAP7000 ICP-OES machine (Thermo Fisher Scientific). Calibration of the equipment was performed by using standard solutions made via dilution of a stock solution of elemental Fe (1 mg/mL). The protein sample was first mixed with 100 μL of concentrated sulfuric acid (H_2SO_4) and 100 μL of concentrated nitric acid (HNO_3) and subsequently heated for 30 min at 250°C. Such a procedure was repeated until the solution became colorless, followed by cooling the solution to room temperature, and diluting the solution to a total volume of 7.5 mL with 2% HNO_3 before metal analysis.

Substrate reduction assays

The substrate reduction assays were performed at 30°C in 9.5-mL vials (DWK Life Science, Millville, NJ). The reaction contained, in a total volume of 1 mL, 25 mM Tris-HCl (pH 8.0), 2.5 mM ATP, 5.0 mM $MgCl_2$, 30 mM creatine phosphate, 0.125 mg of creatine phosphokinase, and 0.5 mM dithionite ($Na_2S_2O_4$); additionally, it contained 0.5 atm C_2H_2/0.5 atm Ar (for C_2H_2-reduction assays), 1.0 atm Ar (for H^+-reduction assays), or 1.0 atm N_2 (for N_2-reduction assays) in the headspace. The reaction was initiated by the addition of 0.15 mg of NifEN and 1.75 mg of NifH, followed by incubation at 30°C for 20 min, quenching with EDTA, and analysis of product formation.

To detect C_2H_4 as a product of C_2H_2-reduction, 250 μL of the headspace was injected into a Gas Chromatography-Flame Ionization Detector (GC-FID) (SRI Instruments, Torrance, CA) equipped with a packed Poropak N column (Restek, Bellefonte, PA). Calibration was achieved by injecting 15-ppm C_2H_4 gas standard under the same conditions. To detect H_2 as the product of H^+-reduction under Ar or N_2, 250 μL of the headspace was injected into a Gas Chromatography-Thermal Conductivity Detector (GC-TCD) (SRI Instruments, Torrance, CA) equipped with a packed Molecular Sieve 5A BOND PLOT column (Restek, Bellefonte, PA). Calibration was achieved by injecting 0.1% H_2 gas standard under the same conditions. To detect NH_3 as the product of N_2-reduction, 100 μL of the reaction was added to a solution that contained, in a total volume of 1 mL, 50 mM potassium phosphate (pH 7.8), 10 mM o-phthalaldehyde (OPA), and 2.5 mM 2-mercaptomethanol. The mixture was allowed to sit at room temperature for 3 h, followed by measurement using a fluorescence spectrophotometer (RF-5301PC, SHIMADZU Co., Ltd., Japan) with the excitation wavelength set at 361 nm and the emission wavelength set at 423 nm.

EPR experiments

Individual EPR samples were prepared in a Vacuum Atmospheres glove box filled with Ar and operated at <3 ppm O_2, and flash frozen in liquid nitrogen before analysis. The reduced samples contained 10% (v/v) glycerol, 250 mM imidazole, 2 mM $Na_2S_2O_4$, and 25 mM tris-

HCl (pH 8.0); the oxidized samples were prepared by incubating the reduced samples with excess indigo disulfonate (IDS) for 5 min. The concentration of the reduced or oxidized sample was 15 mg/mL. EPR data were acquired using an ESP 300E spectrophotometer (Bruker) interfaced with an ESR-9002 liquid-helium continuous-flow cryostat (Oxford Instruments), with a microwave power of 5 mW, a gain of 5×10^4, a modulation frequency of 100 kHz, and a modulation amplitude of 5 G. A total of 8 scans of perpendicular-mode EPR spectra were recorded for each sample at 10 K (for the reduced and oxidized NifEN samples) and 15 K (for the oxidized NifEN samples), respectively, using a microwave frequency of 9.62 GHz; and 16 scans of parallel-mode EPR spectra were recorded for each sample at 10 K (for the oxidized NifEN samples), using a microwave frequency of 9.38 GHz.

NanoSIMS analysis

E. coli strains MY21, YM600EE, YM602EE, YM710EE, and YM713EE, each co-expressing NifH and either wild-type or variant NifEN as a two-component nitrogenase analog, were grown in 100 mL of supplemented M9 medium containing 0.4% glucose, 2 mM $MgSO_4$, 0.1 mM $CaCl_2$, Na_2HPO_4 (12.8 mg/mL), KH_2PO_4 (3 mg/mL), NaCl (0.5 mg/mL), 2 mM NH_4Cl, $FeCl_3$ (0.054 mg/mL), 1 mM cysteine, kanamycin (19 mg/L), and streptomycin (26 mg/L) in 250-mL screw-capped Erlenmeyer flasks with septum-capped side arms for 10 h. Subsequently, the gas phases were exchanged into 100% $^{15}N_2$, followed by addition of 0.5 mM IPTG to each culture, and continued cell growth for another 12 h.

For nanoSIMS analysis, a 250-µL aliquot of each culture (diluted to the same OD) was pipetted onto a 7 mm–by–7 mm silicon square dice cut from wafers (UniversityWafer, Boston, MA) with a diameter of 2.5 cm. The samples were subsequently (i) fixed with a phosphate-buffered saline (PBS) solution containing 4% formaldehyde for 1 h at room temperature; (ii) washed sequentially with PBS, 1:1 PBS/ethanol, and ethanol; and (iii) dried on the wafer.[6] The secondary ion ($^{14}N^{12}C^-$ and $^{15}N^{12}C^-$) and secondary electron images were acquired with the CAMECA NanoSIMS 50L ion microprobe at Caltech (Pasadena, CA). A +8-keV primary Cs^+ beam of ~1 pA was used to raster the samples in 20 µm–by–20 µm areas. Secondary ion ($^{14}N^{12}C^-$ and $^{15}N^{12}C^-$) images of −8 keV were collected simultaneously with electron multiplier detectors. The interferences (*e.g.*, $^{13}C^{13}C^-$ to $^{14}N^{12}C^-$, $^{14}N^{13}C^-$ to $^{15}N^{12}C^-$) were fully removed from the masses of interest under the high mass resolution conditions of the mass spectrometer. Ion images of 512 by 512 pixels were processed with the L'image software (http://limagesoftware.net/).

NMR analysis

Frequency-selective ^1H pulse NMR analysis was performed to assess ATP-dependent reduction of N_2 by wild-type or variant NifEN. Each assay contained, in a total volume of 1.5 mL, 25 mM Tris-HCl (pH 8.0), 0.5 mg of NifEN, 5 mg of NifH, 3.8 mg of YfhL, 5 mM ATP, 52.5 mM $MgCl_2$, 300 mM phosphocreatine, 2 mg of phosphocreatine kinase, and 10 mM sodium dithionite. The reaction mixture was incubated at 30°C for 30 min under 1.0 atm N_2. This step was followed by transfer of the mixture to a 1.5 mL tube containing a Microcon centrifugal filter with a molecular weight cut-off of 10 kDa (Millipore) and removal of proteins by centrifugation at 10,000 g for 20 min. Subsequently, 0.5 mL of the flowthrough was combined with 0.05 mL of 1 M H_2SO_4 and 0.05 mL of CD_3CN as a locking agent. All samples also contained 1 mM maleic acid as an internal reference for signal intensities. The ^1H NMR spectra were recorded using a Bruker AVANCE600 spectrometer equipped with a CBBFO cryoprobe. Water suppression was employed, and the spectra were referenced by setting the residual CH_3CN signal to 2.06 ppm.[7] A total of 256 scans were recorded per sample, with an acquisition time of 1.5 s and a relaxation delay of 5 s.

Boltz-2.2.1 Modeling

The engineered variant, $NifEN^{P/M\_S2B}$, was analyzed using Boltz-2.2.1[8,9] on the Tamarind Bio server (https://www.tamarind.bio/) with a CUDA-enabled NVIDIA A100 80 GB PCIe GPU equipped with Tensor Cores. Amino acid sequences of NifE and NifN of *A. vinelandii* were obtained from the JGI webpage (https://img.jgi.doe.gov/), modified as described for $NifEN^{P/M\_S2B}$ in Table S1, and provided in FASTA format. The M- and P-clusters were provided as CCD codes ICS, HCA and CLF. Template-guided structural prediction was performed by using the mmCIF files of PDB entry 6VXT[10] and applying a template distance threshold of 3 Å. Boltz-2.2.1 was run in protein-ligand mode with default diffusion parameters (200 steps, step scale=1.638) and three recycling iterations. The predicted models were generated and analyzed for the overall confidence scores. The quality of the structure was assessed by visual inspection and through evaluation of cluster coordination geometry. The models exhibit high confidence values, with a very strong overall score of 0.894.

Supporting Figures

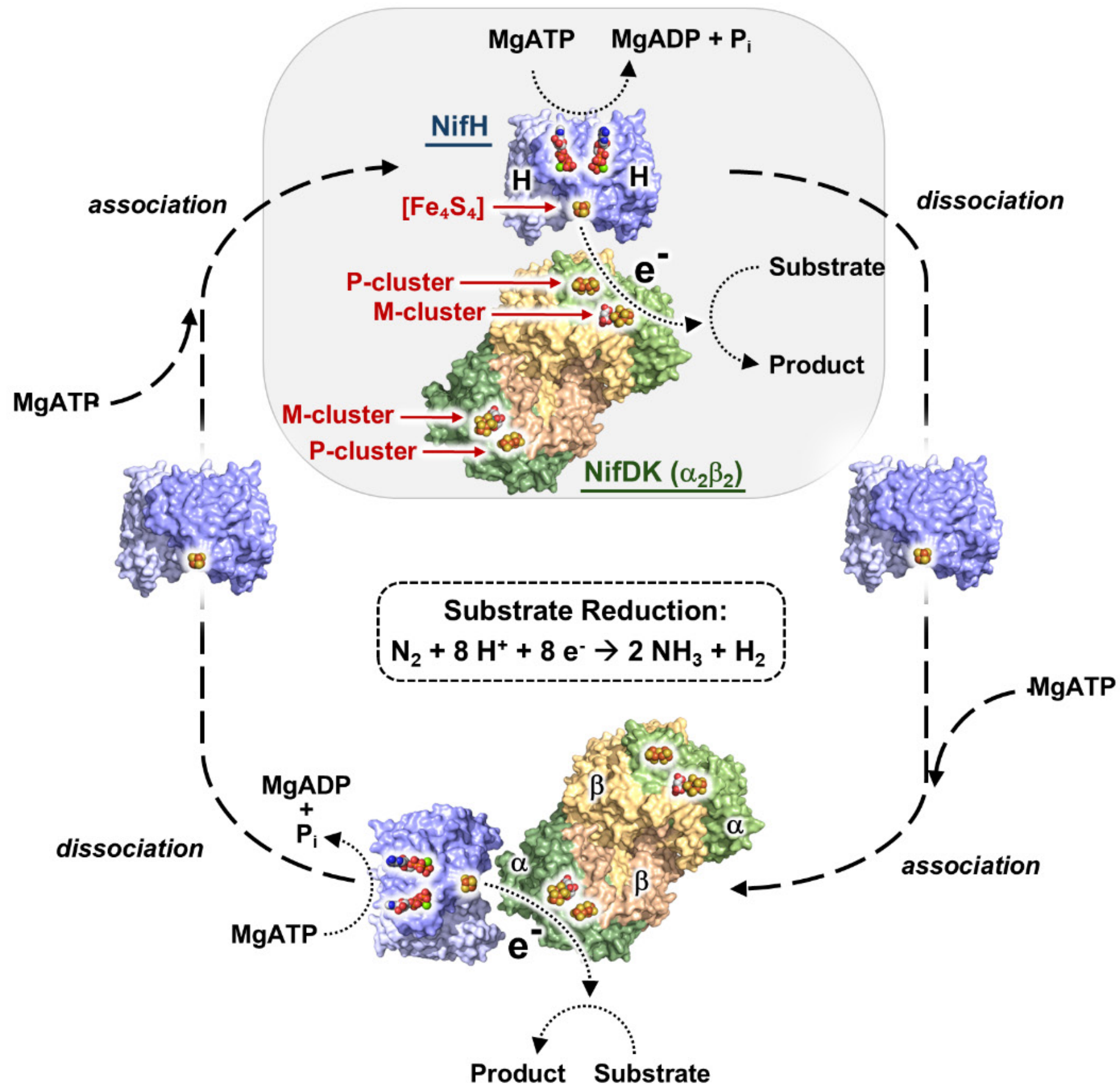

FIGURE S1. Structure and function of the canonical Mo-nitrogenase. The Mo-nitrogenase is a two-component system comprising a homodimeric reductase component (NifH; blue/purple) and an $\alpha_2\beta_2$ heterotetramer catalytic component (NifDK; green/yellow). NifH contains a subunit-bridging $[Fe_4S_4]$ cluster and two MgATP-binding sites; whereas NifDK houses a P-cluster ($[Fe_8S_7]$) at each α/β-subunit interface and an M-cluster ($[(R$-homocitrate$)MoFe_7S_9C]$) within each α-subunit. The NifH/NifDK catalytic cycle involves repeated cycles of MgATP-dependent association and dissociation between NifH and NifDK, driving the transfer of electrons (e^-) from the $[Fe_4S_4]$ cluster of NifH, through the P-cluster, to the M-cluster of NifDK. The overall stoichiometry of the reaction catalyzed by Mo-nitrogenase is usually depicted as follows: $N_2 + 8 H^+ + 8 e^- \rightarrow 2 NH_3 + H_2$. The proteins and metal centers are depicted and colored as described in Figure 1. PyMOL was used to generate this figure, using PDB entry 7UT8.[11]

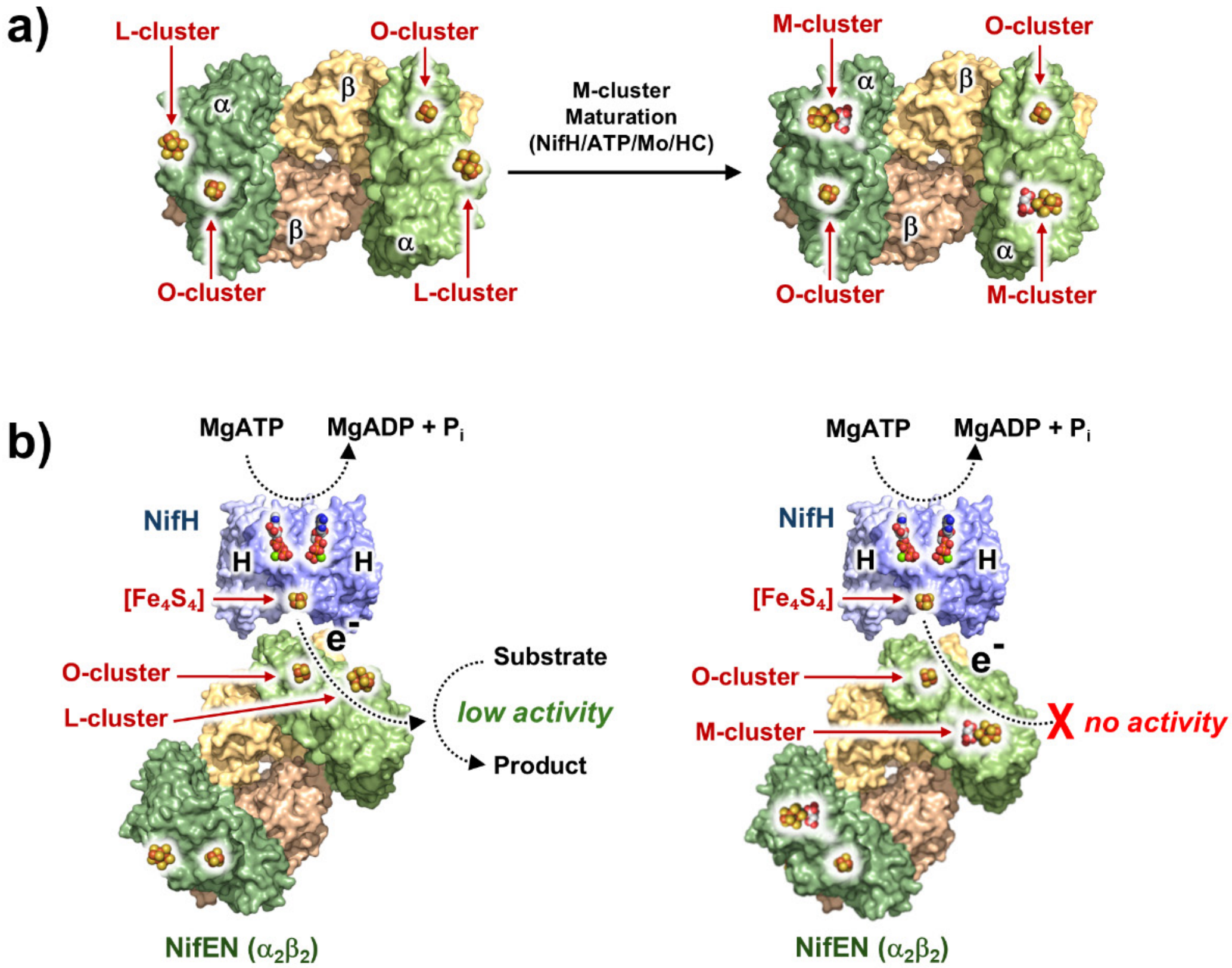

FIGURE S2. Impact of cofactor maturation and relocation on the catalytic capacity of NifEN. (a) Schematic presentation of cofactor maturation and relocation on NifEN. Conversion of the L-cluster ($[Fe_8S_9C]$) into a mature M-cluster ($[(R\text{-homocitrate})MoFe_7S_9C]$) on the $\alpha_2\beta_2$-heterotetrameric NifEN scaffold (green/yellow) at protein surface is enabled by NifH-mediated insertion of Mo and homocitrate (HC) into the L-cluster in an ATP-dependent process. This process is accompanied by the proposed relocation of the M-cluster from the surface-accessible maturation site to an interior cofactor-binding site within the *α*-subunit.[12] **(b)** Functional consequences of cofactor maturation in native NifEN. When loaded with a surface-accessible L-cluster, NifEN exhibits NifH/ATP-dependent catalytic activity (left).[13,14] However, compared with NifDK, the catalytic activity of NifEN is substantially lower and its substrate range is considerably more limited. Such reduced performance in catalysis is likely attributable to the presence of an O-cluster ($[Fe_4S_4]$) rather than the P-cluster ($[Fe_8S_7]$), and an L-cluster ($[Fe_8S_9C]$) rather than the M-cluster ($[(R\text{-homocitrate})MoFe_7S_9C]$), which collectively restrict electron transfer and diminish catalytic efficiency. Upon maturation of the L-cluster into an M-cluster, the mature cofactor is relocated to an interior binding site, accompanied by a concomitant loss of catalytic activity in NifEN.[15,16] The complete loss of catalytic function upon cluster relocation highlights not only the diminished electron transfer resulting from the presence of an "imperfect" O-cluster in NifEN, but also the structural deficiencies of the NifEN interior cofactor environment, which lacks the essential ligands and intact belt region required to support efficient substrate reduction. The proteins and metal centers are depicted and colored as described in Figure 1. PyMOL was used to generate this figure, using PDB entries 7UT8[11] and 3PDI.[17]

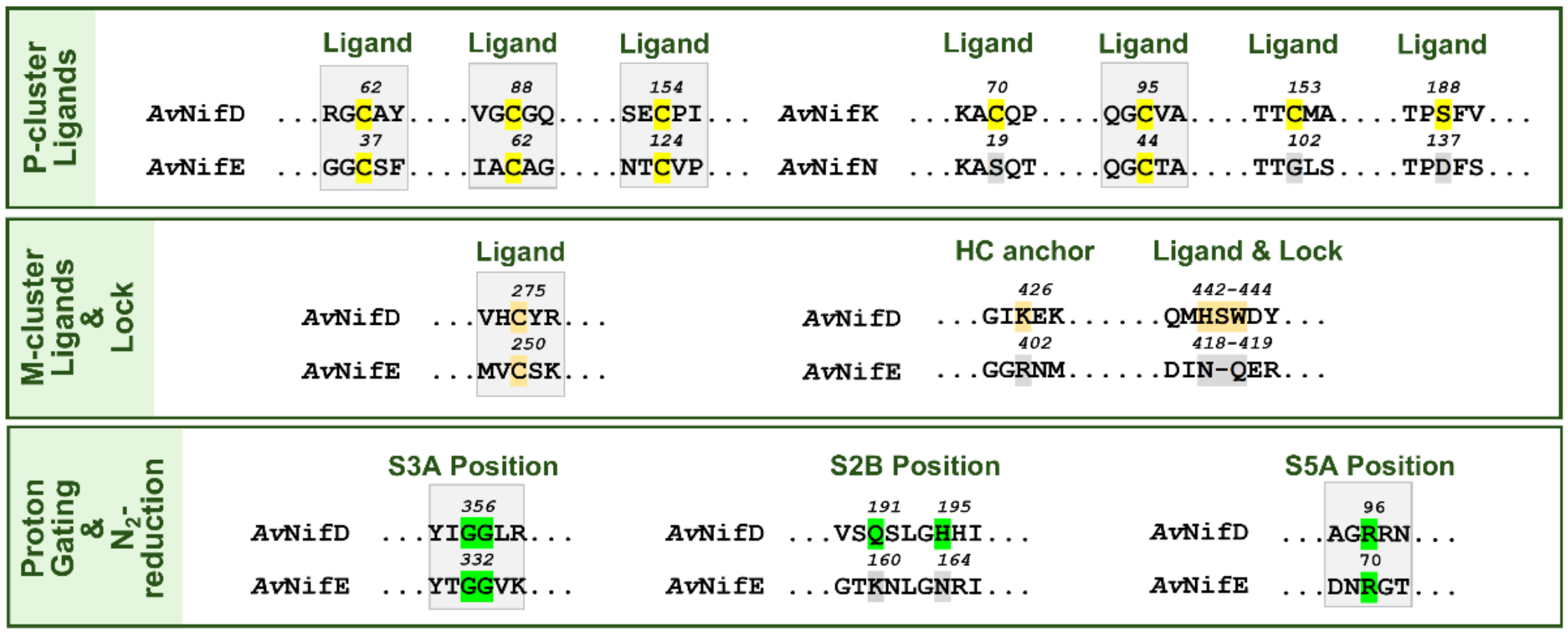

FIGURE S3. Amino acid sequence alignment of NifDK and NifEN in the key regions shown in Figure 1. HC, homocitrate

a)

```
                                                                                                                   S2B Position
                                                                                                                    160   164
NifE^O/M          1   MKAKDIAELLDEPACSHNKKEKSGCAKPKPGATDGGCSFDGAQIALLPVADVAHIVHGPIACAGSSWDNRGTRSSGPDLYRIGMTTDLTE
NifE^P/M_ΔS2B     1   MKAKDIAELLDEPACSHNKKEKSGCAKPKPGATDGGCSFDGAQIALLPVADVAHIVHGPIACAGSSWDNRGTRSSGPDLYRIGMTTDLTE
NifE^P/M_S2B      1   MKAKDIAELLDEPACSHNKKEKSGCAKPKPGATDGGCSFDGAQIALLPVADVAHIVHGPIACAGSSWDNRGTRSSGPDLYRIGMTTDLTE

NifE^O/M         91   NDVIMGRAEKRLFHAIRQAVESYSPPAVFVYNTCVPALIGDDVDAVCKAAAERFGTPVIPVDSAGFYGTKNLGNRIAGEAMLKYVIGTRE
NifE^P/M_ΔS2B    91   NDVIMGRAEKRLFHAIRQAVESYSPPAVFVYNTCVPALIGDDVDAVCKAAAERFGTPVIPVDSAGFYGTKNLGNRIAGEAMLKYVIGTRE
NifE^P/M_S2B     91   NDVIMGRAEKRLFHAIRQAVESYSPPAVFVYNTCVPALIGDDVDAVCKAAAERFGTPVIPVDSAGFYGTQNLGHRIAGEAMLKYVIGTRE

NifE^O/M        181   PDPLPVGSERPGIRVHDVNLIGEYNIAGEFWHVLPLLDELGLRVLCTLAGDARYREVQTMHRAEVNMMVCSKAMLNVARKLQETYGTPWF
NifE^P/M_ΔS2B   181   PDPLPVGSERPGIRVHDVNLIGEYNIAGEFWHVLPLLDELGLRVLCTLAGDARYREVQTMHRAEVNMMVCSKAMLNVARKLQETYGTPWF
NifE^P/M_S2B    181   PDPLPVGSERPGIRVHDVNLIGEYNIAGEFWHVLPLLDELGLRVLCTLAGDARYREVQTMHRAEVNMMVCSKAMLNVARKLQETYGTPWF

NifE^O/M        271   EGSFYGITDTSQALRDFARLLDDPDLTARTEALIAREEAKVRAALEPWRARLEGKRVLLYTGGVKSWSVVSALQDLGMKVVATGTKKSTE
NifE^P/M_ΔS2B   271   EGSFYGITDTSQALRDFARLLDDPDLTARTEALIAREEAKVRAALEPWRARLEGKRVLLYTGGVKSWSVVSALQDLGMKVVATGTKKSTE
NifE^P/M_S2B    271   EGSFYGITDTSQALRDFARLLDDPDLTARTEALIAREEAKVRAALEPWRARLEGKRVLLYTGGVKSWSVVSALQDLGMKVVATGTKKSTE

                                                                   HC Anchor     M-cluster ligand & Lock
                                                                      402              418
NifE^O/M        361   EDKARIRELMGDDVKMLDEGNARVLLKTVDEYQADILIAGGRNMYTALKGRVPFLDIN-QEREFGYAGYDGMLELVRQLCITLECPVWEA
NifE^P/M_ΔS2B   361   EDKARIRELMGDDVKMLDEGNARVLLKTVDEYQADILIAGGKNMYTALKGRVPFLDIHSWEREFGYAGYDGMLELVRQLCITLECPVWEA
NifE^P/M_S2B    361   EDKARIRELMGDDVKMLDEGNARVLLKTVDEYQADILIAGGKNMYTALKGRVPFLDIHSWEREFGYAGYDGMLELVRQLCITLECPVWEA

NifE^O/M        450   VRRPAPWDIPASQDAAPSAPARSANA
NifE^P/M_ΔS2B   451   VRRPAPWDIPASQDAAPSAPARSANA
NifE^P/M_S2B    451   VRRPAPWDIPASQDAAPSAPARSANA
```

FIGURE S4. Sequence alignments of NifEN variants engineered in this study. Amino acid sequence alignments of the **(a)** NifE and **(b)** NifN subunits of the NifEN variants listed in Table S1. HC, homocitrate

b)

P-cluster ligand 19

$NifN^{O/M}$ 1 MAEIINRNKALAVSPLKASQTMGAALAILGLARSMPLFHGSQGCTAFAKVFFVRHFREPVPLQTTAMDQVSSVMGADENVVEALKTICER
$NifN^{P/M\_\Delta S2B}$ 1 MAEIINRNKALAVSPLKACQTMGAALAILGLARSMPLFHGSQGCTAFAKVFFVRHFREPVPLQTTAMDQVSSVMGADENVVEALKTICER
$NifN^{P/M\_S2B}$ 1 MAEIINRNKALAVSPLKACQTMGAALAILGLARSMPLFHGSQGCTAFAKVFFVRHFREPVPLQTTAMDQVSSVMGADENVVEALKTICER

P-cluster ligand 102 P-cluster ligand 137

$NifN^{O/M}$ 91 QNPSVIGLLTTGLSETQGCDLHTALHEFRTQYEEYKDVPIVPVNTPDFSGCFESGFAAAVKAIVETLVPERRDQVGKRPRQVNVLCSANL
$NifN^{P/M\_\Delta S2B}$ 91 QNPSVIGLLTTCLSETQGCDLHTALHEFRTQYEEYKDVPIVPVNTPSFSGCFESGFAAAVKAIVETLVPERRDQVGKRPRQVNVLCSANL
$NifN^{P/M\_S2B}$ 91 QNPSVIGLLTTCLSETQGCDLHTALHEFRTQYEEYKDVPIVPVNTPSFSGCFESGFAAAVKAIVETLVPERRDQVGKRPRQVNVLCSANL

$NifN^{O/M}$ 181 TPGDLEYIAESIESFGLRPLLIPDLSGSLDGHLDENRFNALTTGGLSVAELATAGQSVATLVVGQSLAGAADALAERTGVPDRRFGMLYG
$NifN^{P/M\_\Delta S2B}$ 181 TPGDLEYIAESIESFGLRPLLIPDLSGSLDGHLDENRFNALTTGGLSVAELATAGQSVATLVVGQSLAGAADALAERTGVPDRRFGMLYG
$NifN^{P/M\_S2B}$ 181 TPGDLEYIAESIESFGLRPLLIPDLSGSLDGHLDENRFNALTTGGLSVAELATAGQSVATLVVGQSLAGAADALAERTGVPDRRFGMLYG

$NifN^{O/M}$ 271 LDAVDAWLMALAEISGNPVPDRYKRQRAQLQDAMLDTHFMLSSARTAIAADPDLLLGFDALLRSMGAHTVAAVVPARAAALVDSPLPSVR
$NifN^{P/M\_\Delta S2B}$ 271 LDAVDAWLMALAEISGNPVPDRYKRQRAQLQDAMLDTHFMLSSARTAIAADPDLLLGFDALLRSMGAHTVAAVVPARAAALVDSPLPSVR
$NifN^{P/M\_S2B}$ 271 LDAVDAWLMALAEISGNPVPDRYKRQRAQLQDAMLDTHFMLSSARTAIAADPDLLLGFDALLRSMGAHTVAAVVPARAAALVDSPLPSVR

$NifN^{O/M}$ 361 VGDLEDLEHAARAGQAQLVIGNSHALASARRLGVPLLRAGFPQYDLLGGFQRCWSGYRGSSQVLFDLANLLVEHHQGIQPYHSIYAQKPA
$NifN^{P/M\_\Delta S2B}$ 361 VGDLEDLEHAARAGQAQLVIGNSHALASARRLGVPLLRAGFPQYDLLGGFQRCWSGYRGSSQVLFDLANLLVEHHQGIQPYHSIYAQKPA
$NifN^{P/M\_S2B}$ 361 VGDLEDLEHAARAGQAQLVIGNSHALASARRLGVPLLRAGFPQYDLLGGFQRCWSGYRGSSQVLFDLANLLVEHHQGIQPYHSIYAQKPA

$NifN^{O/M}$ 451 TEQPQWRH
$NifN^{P/M\_\Delta S2B}$ 451 TEQPQWRH
$NifN^{P/M\_S2B}$ 451 TEQPQWRH

FIGURE S4. (continued)

a)

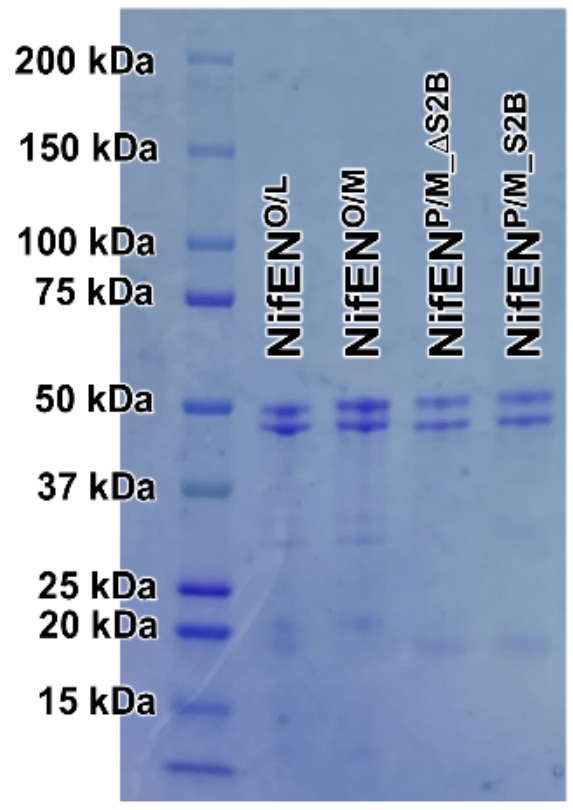

b)

Protein	Mo content (mol/mol NifEN)	Fe content (mol/mol NifEN)	M-cluster per NifEN		L-cluster per NifEN		P-cluster per NifEN		$[Fe_4S_4]$ Per NifEN	
			based on Mo content	*calculated Fe content of M-cluster*	*based on EPR*	*calculated Fe content of L-cluster*	*based on EPR*	*calculated Fe content of P-cluster*	*remaining Fe content*	*based on Fe content*
NifEN$^{O/L}$	>0.01	19.1 ± 2.0	0	0	1.4	11.2	0	0	7.9	2
NifEN$^{O/M}$	1.02 ± 0.23	15.5 ± 1.4	1.02	7.1	0	0	0	0	8.4	2.1
NifEN$^{P/M_\Delta S2B}$	0.55 ± 0.08	11.8 ± 1.9	0.55	3.9	0	0	0.44	3.5	4.4	1.1
NifEN$^{P/M_S2B}$	0.38 ± 0.05	10.7 ± 1.5	0.38	2.7	0	0	0.68	5.4	2.6	0.7

FIGURE S5. Subunit and cluster compositions of various NifEN proteins. **(a)** SDS-PAGE analysis and **(b)** cluster assignment of NifEN$^{O/L}$, NifEN$^{O/M}$, NifEN$^{P/M_\Delta S2B}$ and NifEN$^{P/M_S2B}$. The M-cluster content of each protein was determined from its Mo content, assuming one Mo atom per M-cluster based on the cluster composition ([(*R*-homocitrate)$MoFe_7S_9C$]). The Fe contribution of the M-cluster was calculated assuming seven Fe atoms per M-cluster based on the cluster composition. The L-cluster content was determined from the intensity of the L-cluster-specific *g*=1.95 EPR signal in the IDS-oxidized state (*see* Figure 2c), using *Azotobacter vinelandii* NifEN, which contains two L-clusters per protein, as the reference. The Fe contribution of the L-cluster was calculated assuming eight Fe atoms per L-cluster, based on the cluster composition ([Fe_8S_9C]). The P-cluster content was determined from the intensity of the P^{OX}-specific *g*≈12 EPR signal in the IDS-oxidized state (*see* Figure 2a), using *Azotobacter vinelandii* NifDK, which contains two P-clusters per protein, as the reference. The percentage of P-clusters in Figure 2a was calculated by dividing the P-cluster content by the theoretical maximum occupancy of two P-clusters per protein. The Fe contribution of the P-cluster was calculated assuming eight Fe atoms per P-cluster based on the cluster composition ([Fe_8S_7]). The remaining Fe content, obtained by subtracting the Fe contributions of the M-, L-, and P-clusters from the total Fe content, was assigned to [Fe_4S_4]-type clusters (*i.e.*, the O-cluster or the immature P*-cluster comprising paired [Fe_4S_4] clusters). This assignment is consistent with the presence of *S*=1/2 EPR signals in the dithionite-reduced proteins (*see* Figure 2b), which are characteristic of [Fe_4S_4]-type clusters.

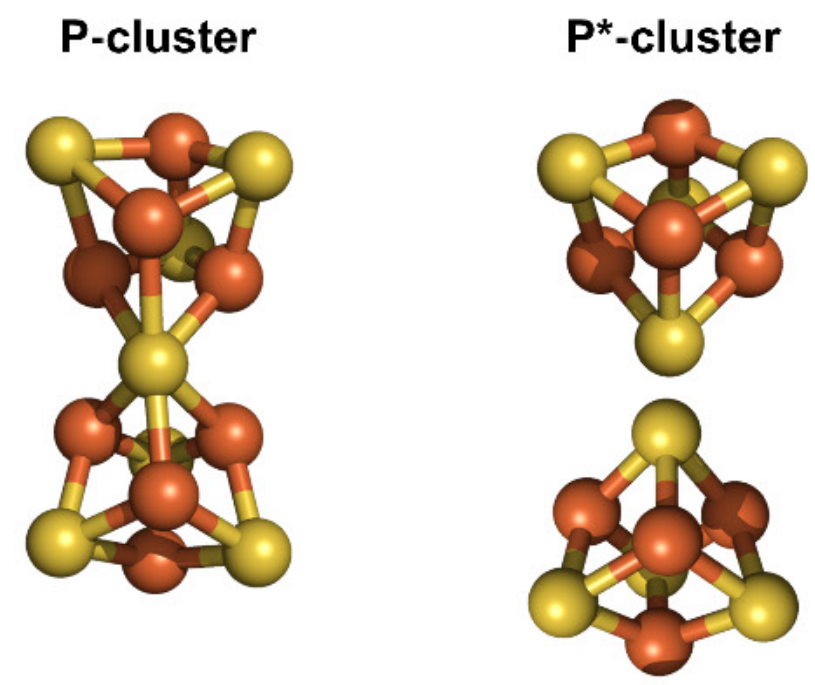

FIGURE S6. Structural comparison of the P-cluster and P*-cluster (P-cluster precursor). The structure of the P-cluster is based on the published crystal structure (PDB entry 7UT8[11]), whereas the structure of the P-cluster precursor is derived from XAS/EXAFS-based structural modeling.[19]

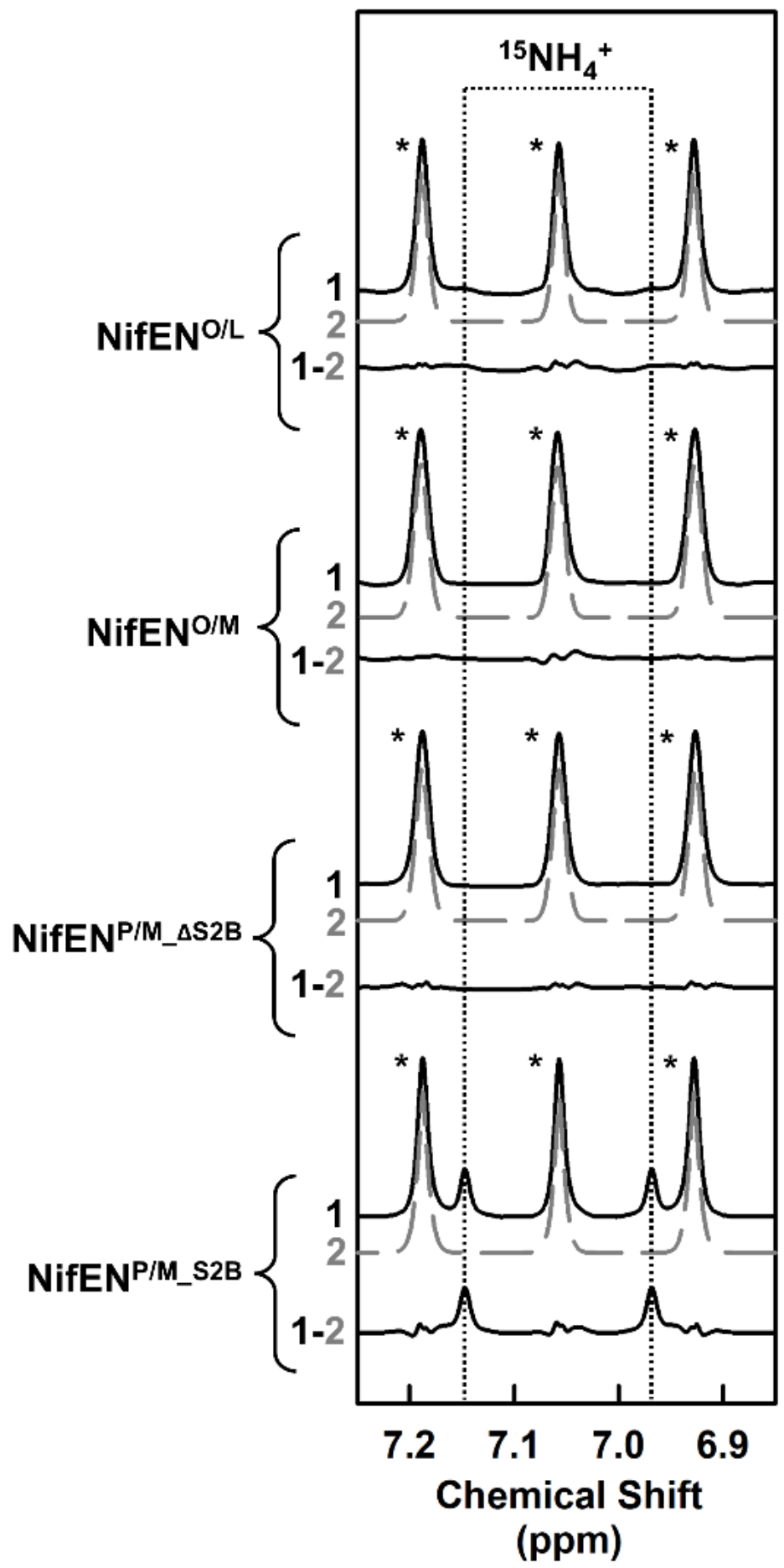

FIGURE S7. Frequency-selective pulse ^{1}H NMR spectra of $^{15}N_2$ reduction by NifEN$^{O/L}$, NifEN$^{O/M}$, NifEN$^{P/M_\Delta S2B}$, and NifEN$^{P/M_S2B}$ in *in vitro* assays driven by NifH, ATP, and dithionite. Shown are (1) the recorded assay spectra; (2) a three-peak Gaussian simulation of the natural-abundance $^{14}NH_4^+$ background (indicated by *); and (3) the difference spectra obtained by subtracting (2) from (1), which reveal the $^{15}NH_4^+$-specific doublet at ~6.97 and ~7.15 ppm in the spectrum of NifEN$^{P/M_S2B}$, arising from the reduction of $^{15}N_2$ to $^{15}NH_4^+$.

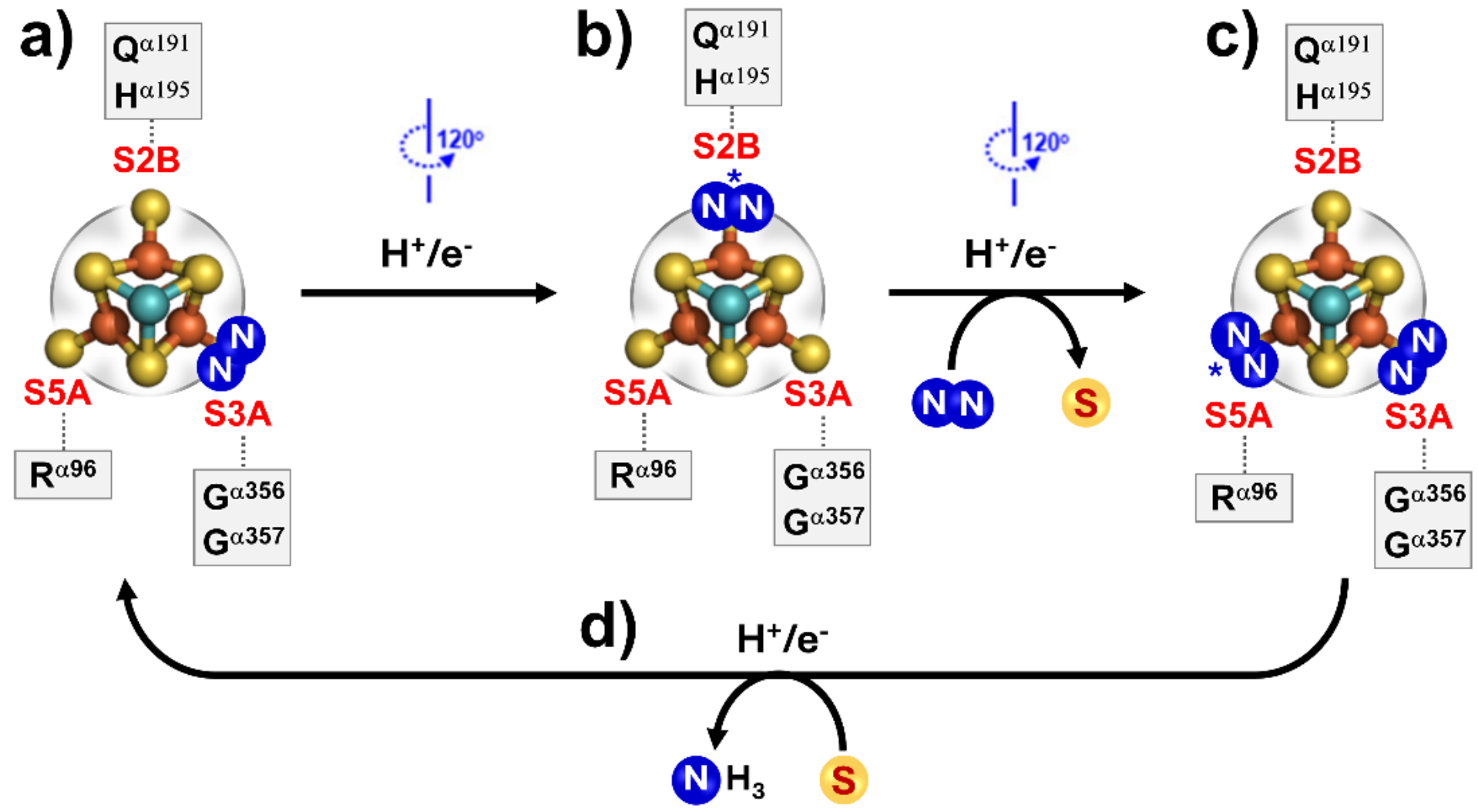

FIGURE S8. Proposed model for stepwise N_2 reduction at the three belt-sulfur sites via M-cluster rotation.[10] Rotation of the M-cluster in the direction of S3A→S2B→S5A is proposed to be enabled by alternating elongation and protonation-induced cleavage of the two Mo–O bonds linking the Mo center to homocitrate. This rotational motion permits N_2 binding at the S3A site through displacement of the belt sulfur (**a**), followed by stepwise reduction of the substrate to NH_3 at the S2B (**b**) and S5A (**c**) sites. Binding of the next N_2 molecule at the regenerated S3A site via belt-sulfur displacement (**c**) is accompanied by the release of NH_3 from the S5A site through belt-sulfur re-incorporation (**d**). Continuous M-cluster rotation is proposed to couple these events, enabling successive cycles of N_2 binding, reduction, and product release.

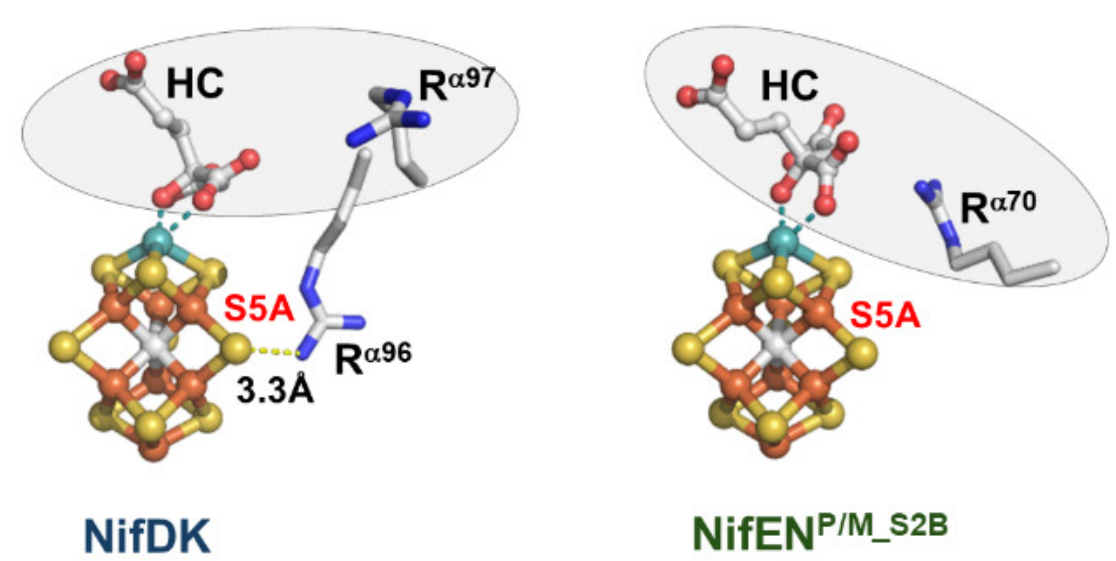

FIGURE S9. Structural comparison of the S5A sites in NifDK and NifEN$^{P/M_S2B}$. The native $R^{\alpha70}$ in NifEN$^{P/M_S2B}$ is expected to mimic $R^{\alpha96}$ in NifDK based on amino acid sequence alignment (*see* Figure S3). However, Boltz-2.2.1 predicts that this residue occupies a position structurally analogous to $R^{\alpha97}$ in NifDK, where it interacts with the homocitrate ligand and helps organize the local hydrogen-bonding network, leaving the $R^{\alpha96}$ equivalent absent from NifEN$^{P/M_S2B}$. The metal centers are depicted and colored as described in Figure 1. PyMOL was used to generate this figure, using PDB entry 3U7Q[18] for NifDK, and Boltz-2.2.1 prediction for NifEN$^{P/M_S2B}$.

Supporting Table

TABLE S1. *E. coli* strains and engineered proteins used in this study

Strain	Parent strain	Genome deletion	Vectors (Genes)	Site-directed mutations	Expected cluster composition (Cell growth with Mo)	NifEN designation	Schematic presentation*
YM710EE	MY21[2]	Δ*iscR*	<u>pRSFDuet-1:</u><br>*Ma_nifU3, S3, B*<br>*Av_nifH, M, V*<br><br><u>pCDFDuet-1:</u><br>*Ma_nifU3, S3, B*<br>*Av_nifE*$^{His\text{-}tagged}$, *N*		<u>O-cluster</u><br><br><u>M-cluster</u>:<br>no homocitrate (HC) anchor,<br>no His-ligand/lock,<br>impaired S2B site	**NifEN$^{O/M}$**	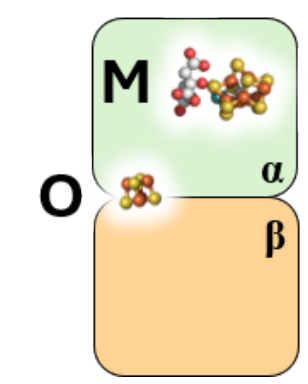
YM600EE	MY21[2]	Δ*iscR*	<u>pRSFDuet-1:</u><br>*Ma_nifU3, S3, B*<br>*Av_nifH, M, Z, V*<br><br><u>pCDFDuet-1:</u><br>*Ma_nifU3, S3, B*<br>*Av_nifE*$^{*,\ His\text{-}tagged}$, *N**	<u>P-cluster ligands:</u><br>NifN: S19C, G102C, D137S<br><br><u>M-cluster ligands and Lock:</u><br>NifE: R402K, N418H,419S^{ins}(*ins*, inserted), Q420W	<u>P-cluster:</u><br>all ligands restored<br><br><u>M-cluster</u>:<br>HC anchor restored,<br>His-ligand/lock restored,<br>impaired S2B site	**NifEN$^{P/M_\Delta S2B}$**	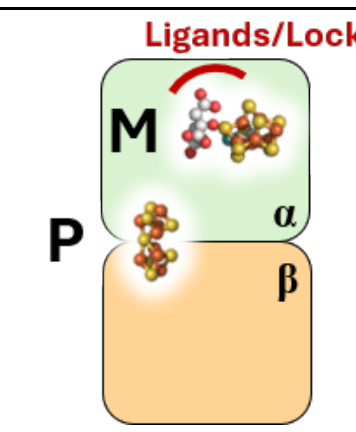
YM602EE	MY21[2]	Δ*iscR*	<u>pRSFDuet-1:</u><br>*Ma_nifU3, S3, B*<br>*Av_nifH, M, Z, V*<br><br><u>pCDFDuet-1:</u><br>*Ma_nifU3, S3, B*<br>*Av_nifE*$^{*,\ His\text{-}tagged}$, *N**	<u>P-cluster ligands:</u><br>NifN: S19C, G102C, D137S<br><br><u>M-cluster ligands and Lock:</u><br>NifE: R402K, N418H, 419S^{ins}, Q420W<br><br><u>S2B position:</u><br>NifE: K160Q, N164H	<u>P-cluster:</u><br>all ligands restored<br><br><u>M-cluster</u>:<br>HC anchor restored,<br>His-ligand/lock restored,<br>S2B site restored	**NifEN$^{P/M_S2B}$**	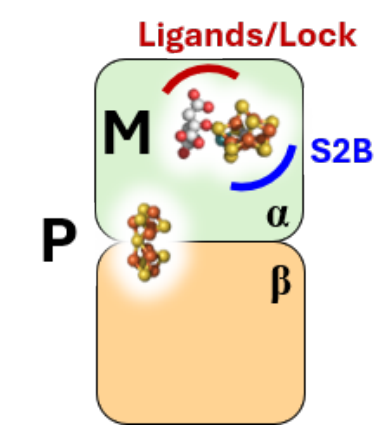
YM713EE	MY25[1]	Δ*iscR*,<br>Δ*yfhL*	<u>pRSFDuet-1:</u><br>*Ma_nifU3, S3, B*<br>*Av_nifH, M, Z, V*<br><br><u>pCDFDuet-1:</u><br>*Ma_nifU3, S3, B*<br>*Av_nifE*$^{*,\ His\text{-}tagged}$, *N**	<u>P-cluster ligands:</u><br>NifN: S19C, G102C, D137S<br><br><u>M-cluster ligands and Lock:</u><br>NifE: R402K, N418H, 419S^{ins}, Q420W<br><br><u>S2B position:</u><br>NifE: K160Q, N164H	<u>P-cluster:</u><br>all ligands restored<br><br><u>M-cluster</u>:<br>HC anchor restored,<br>His-ligand/lock restored,<br>S2B site restored	**NifEN$^{P/M_S2B}$**	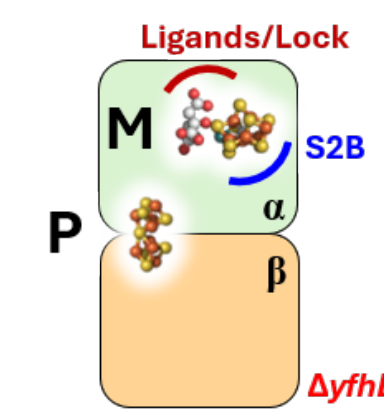

* Only one αβ-dimer is shown for the purpose of clarity.

References

[1] Y. A. Liu, C. C. Lee, K. Górecki, M. T. Stiebritz, C. Duffin, J. B. Solomon, M. W. Ribbe, Y. Hu, "Heterologous synthesis of a simplified nitrogenase analog in *Escherichia coli*." *Sci. Adv.* **2025**, 11, eadw6785.

[2] J. B. Solomon, C. C. Lee, Y. A. Liu, C. Duffin, M. W. Ribbe, Y. Hu, "Ammonia synthesis via an engineered nitrogenase assembly pathway in *Escherichia coli*." *Nat. Catal.* **2024**, 7, 1130.

[3] Y. Hu, A. W. Fay, M. W. Ribbe, "Identification of a nitrogenase FeMo cofactor precursor on 674 NifEN complex." *Proc. Natl. Acad. Sci. U. S A.* **2005**, 102, 3236.

[4] Y. Hu, A. W. Fay, P. C. Dos Santos, F. Naderi, M. W. Ribbe, "Characterization of *Azotobacter vinelandii nifZ* deletion strains. Indication of stepwise MoFe protein assembly." *J. Biol. Chem.* **2004**, 279, 54963.

[5] M. W. Ribbe, Y. Hu, M. Guo, B. Schmid, B. K. Burgess, "The FeMoco-deficient MoFe protein produced by a *nifH* deletion strain of *Azotobacter vinelandii* shows unusual P-cluster features." *J. Biol. Chem.* **2002**, 277, 23469.

[6] A. Pernthaler, A. E. Dekas, C. T. Brown, S. K. Goffredi, T. Embaye, V. J. Orphan, "Diverse syntrophic partnerships from deep-sea methane vents revealed by direct cell capture and metagenomics." *Proc. Natl. Acad. Sci. U. S. A.* **2008**, 105, 7052.

[7] G. R. Fulmer, A. J. M. Miller, N. H. Sherden, H. E. Gottlieb, A. Nudelman, B. M. Stoltz, J. E. Bercaw, K. I. Goldberg, "NMR chemical shifts of trace impurities: common laboratory solvents, organics, and gases in deuterated solvents relevant to the organometallic chemist." *Organometallics* **2010**, 29, 2176.

[8] S. Passaro, G. Corso, J. Wohlwend, M. Reveiz, S. Thaler, V. R. Somnath, N. Getz, T. Portnoi, J. Roy, H. Stark, D. Kwabi-Addo, D. Beaini, T. Jaakkola, R. Barzilay, "Boltz-2: towards accurate and efficient binding affinity prediction." *bioRxiv* preprint **2025**, DOI: 10.1101/2025.06.14.659707.

[9] J. Wohlwend, G. Corso, S. Passaro, N. Getz, M. Reveiz, K. Leidal, W. Swiderski, L. Atkinson, T. Portnoi, I. Chinn, J. Silterra, T. Jaakkola, R. Barzilay, "Boltz-1 democratizing biomolecular interaction modeling." *bioRxiv* preprint **2025**, DOI: 10.1101/2024.11.19.624167.

[10] W. Kang, C. C. Lee, A. J. Jasniewski, M. W. Ribbe, Y. Hu, "Structural evidence for a dynamic metallocofactor during N_2 reduction by Mo-nitrogenase." *Science* **2020**, 368, 1381.

[11] H. L. Rutledge, B. D. Cook, H. P. M. Nguyen, M. A. Herzik Jr., F. A. Tezcan, "Structures of the nitrogenase complex prepared under catalytic turnover conditions." *Science* **2022**, 377, 865.

[12] A. W. Fay, M. A. Blank, J. G. Rebelein, C. C. Lee, M. W. Ribbe, B. Hedman, K. O. Hodgson, Y. Hu, "Assembly scaffold NifEN: A structural and functional homolog of the nitrogenase catalytic component." *Proc. Natl. Acad. Sci. U. S. A.* **2016**, 113, 9504.

[13] Y. Hu, J. M. Yoshizawa, A. W. Fay, C. C. Lee, J. A. Wiig, M. W. Ribbe, "Catalytic activities of NifEN: implications for nitrogenase evolution and mechanism." *Proc. Natl. Acad. Sci. U. S. A.* **2009**, 106, 16962.

[14] C. C. Lee, K. Góreck, M. Stang, M. W. Ribbe, Y. Hu, "Cofactor maturase NifEN: A prototype ancient nitrogenase?" *Sci. Adv.* **2024**, 10, eado6169.

[15] A. W. Fay, M. A. Blank, J. G. Rebelein, C. C. Lee, M. W. Ribbe, B. Hedman, K. O. Hodgson, Y. Hu, "Assembly scaffold NifEN: A structural and functional homolog of the nitrogenase catalytic component." *Proc. Natl. Acad. Sci. U. S. A.* **2016**, 113, 9504.

[16] J. M. Yoshizawa, A. W. Fay, C. C. Lee, Y. Hu, M. W. Ribbe, "Insertion of heterometals into the NifEN-associated iron-molybdenum cofactor precursor." *J. Biol. Inorg. Chem.* **2010**, 15, 421.

[17] J. T. Kaiser, Y. Hu, J. A. Wiig, D. C. Rees, M. W. Ribbe, "Structure of precursor-bound NifEN: a nitrogenase FeMo cofactor maturase/insertase." *Science* **2011**, 331, 91.

[18] T. Spatzal, M. Aksoyoglu, L. Zhang, S. L. Andrade, E. Schleicher, S. Weber, D. C. Rees, O. Einsle, "Evidence for interstitial carbon in nitrogenase FeMo cofactor." *Science* **2011**, 334, 940.

[19] M. C. Corbett, Y. Hu, F. Naderi, M. W. Ribbe, B. Hedman, K. O. Hodgson, "Comparison of iron-molybdenum cofactor-deficient nitrogenase MoFe proteins by X-ray absorption spectroscopy: implications for P-cluster biosynthesis." *J. Biol. Chem.* **2004**, 279, 28276.
